# Tanhuo Formula Inhibits Astrocyte Activation and Apoptosis in Acute Ischemic Stroke

**DOI:** 10.3389/fphar.2022.859244

**Published:** 2022-04-26

**Authors:** Yuting Nie, Lulu Wen, Hui Li, Juexian Song, Ningqun Wang, Liyuan Huang, Li Gao, Miao Qu

**Affiliations:** ^1^ Department of Neurology, Xuanwu Hospital, Capital Medical University, Beijing, China; ^2^ Capital Medical University, Beijing, China; ^3^ Institute of Chinese Materia Medica, China Academy of Chinese Medical Sciences, Beijing, China

**Keywords:** Tanhuo formula (THF), inflammation, astrocyte, apoptosis, network pharmacology

## Abstract

Tanhuo formula (THF), a traditional Chinese medicinal formula, has been demonstrated to be effective in the clinical treatment of acute ischemic stroke (AIS). However, its active ingredients, potential targets, and molecular mechanisms remain unknown. Based on the validation of active ingredient concentrations, our study attempted to elucidate the possible mechanisms of THF based on network pharmacological analysis and experimental validation. Components of THF were screened using network pharmacological analysis, and a compound–target network and protein–protein interaction (PPI) network were constructed. In total, 42 bioactive compounds and 159 THF targets related to AIS were identified. The PPI network identified AKT1, TNF, IL6, IL1B, and CASP3 as key targets. Kyoto Encyclopedia of Genes and Genomes pathway enrichment analysis demonstrated that the inflammation and apoptotic pathways were enriched by multiple targets. The main components of THF were identified *via* high-performance liquid chromatography. Subsequently, a validation experiment was conducted, and the expressions of GFAP, C3, TNF-α, and IL-6 were detected *via* immunofluorescence staining, confirming the inflammatory response at 30 min and 3 days post injury. Immunohistochemical staining for caspase-3 and TUNEL was also performed to assess apoptosis at the same time points. These results indicate that THF can effectively decrease neural cell apoptosis through the caspase-3 pathway and restrain excessive abnormal activation of astrocytes and the release of TNF-α and IL-6, which might be accompanied by the recovery of motor function. Thus, THF may serve as a promising therapeutic strategy for AIS through multiple targets, components, and pathways.

## Introduction

Acute ischemic stroke (AIS), characterized by high morbidity, disability, and mortality, is one of the leading causes of human death and disability worldwide; it, thereby, imposes an immense financial burden on society and severe psychological stress on individuals (Herpich and Rincon, 2020; Gao et al., 2020). According to the World Health Organization, approximately 15 million people experience an ischemic stroke each year, of which approximately 5 million die (Esquiva et al., 2018; Krishnamurthi et al., 2020; Morovatdar et al., 2021). Recombinant tissue–type plasminogen activator, a unique, FDA-approved drug for the treatment of AIS, can only benefit approximately 5% of patients owing to the narrow treatment window for thrombolysis and the risk of cerebral hemorrhage induced by the drug (Saberi et al., 2021). Therefore, it is essential to develop more effective and safe drugs with minimal side effects for AIS treatment.

Increasing evidence has demonstrated that intervening inflammatory reactions are important targets in the clinical treatment of AIS. Astrocytes are important regulators of the neuroinflammatory response and have also been found to be effective targets for therapeutic intervention in ischemic stroke (Patabendige et al., 2021). Owing to the widespread distribution and intrinsic features of the central nervous system (CNS), astrocytes are rapidly activated and proliferated after ischemic stroke (Stary and Giffard, 2015). Activated astrocytes can immediately differentiate into two polarization phenotypes, A1 and A2. The former has pro-inflammatory and neurotoxic effects, whereas the latter has anti-inflammatory and neural restoration effects. Overactivated A1 astrocytes secrete various types of inflammatory cytokines and a large number of cytotoxic substances, such as ROS, iNOS, and glutamate. These substances amplify the inflammatory cascade reaction, aggravate the redistribution or degradation of key tight junctions (such as claudin-5 and occludin), and deteriorate the leakage of the blood–brain barrier, ultimately causing severe tissue damage and neuronal death (Xu et al., 2020; Qiu et al., 2021; Sun et al., 2021). Apoptosis is the major type of cell death at the location of cerebral infarction (Radak et al., 2017). Neurons are sensitive to the deterioration of the microenvironment and undergo massive apoptosis after a series of ischemia, hypoxia, oxidative stress, and inflammatory responses following AIS (Xu et al., 2020). Thus, inhibition of apoptosis and activation of A1 astrocytes are an essential neuroprotective strategy for AIS.

**FIGURE 1:**
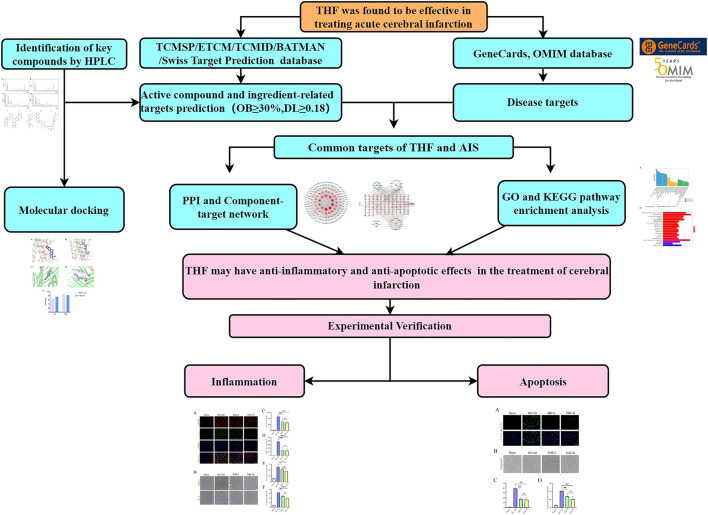
Framework of this study.

For many years, Tanhuo formula (THF), a traditional Chinese medicinal formula, has been generally acknowledged as an effective prescription for AIS caused by phlegm-heat syndrome in Xuanwu Hospital. Although our previous study confirmed that THF reduced infarct volume and attenuated neuronal damage in cerebral ischemic rats, the mechanism of antiapoptosis and astrocyte regulation has not yet been elucidated (Cong et al., 2021). THF comprises five herbal medicines: dried rhizome of *Coptis chinensis Franch.* [*Ranunculaceae*; Coptidis rhizoma; Huang-Lian (HL)], dried roots and rhizomes of *Rheum officinale Baill.*, [*Polygonaceae*, Rhei radix et rhizoma, Da-Huang (DH)], dried fruits of *Forsythia suspensa (Thunb.) Vahl* [*Oleaceae*; Forsythiae fructus; Lian-Qiao (LQ)], dried leaves of *Lophatherum gracile Brongn.* [*Poaceae*; Lophatheri herba; Dan-Zhu-Ye (DZY)], and *Rhizoma Arisaematis Cum Bile* [*Araceae*; Arisaema cum bile; Dan-Nan-Xing (DNX)]. The herbal components of THF, such as aloe emodin in DH, phillyrin in LQ, berberine, and palmatine in HL, have been shown to inhibit inflammatory responses and neuronal apoptosis in the brain (Zhu et al., 2019; Guo X. et al., 2021; Jiang et al., 2021; Tang et al., 2021; Xian et al., 2021; Zhao et al., 2021). We determined the relatively high concentrations of these key components in THF using high-performance liquid chromatography (HPLC). Based on the required concentrations to exert pharmacological effects, it is reasonable to infer that these THF components can exert pharmacological effects in the human body and promote recovery by inhibiting inflammatory response and reducing apoptosis in AIS. However, THF consists of several herbs and contains a variety of components that act *via* multiple pathways and may influence each other. Therefore, we comprehensively investigated the pharmacodynamics of THF on AIS by considering a variety of components and using network pharmacology methods. Based on bioinformatics and systems biology, network pharmacology is used to study the multilevel network relationship of “drug–target–disease” by screening potential pharmacodynamic components, action targets, and pathways in a database. Although the formula for THF has multiple ingredients and targets, network pharmacology can help address this challenge by precisely determining the research target of THF and revealing the underlying mechanisms for AIS effectively. However, current network pharmacology studies focus on the chemical composition of herbal medicines instead of the dose–effect relationship, and the content of compounds also affects the effect of herbal medicines. Therefore, the prediction results of network pharmacology may be biased and need to be experimentally verified (Zhang et al., 2019). Herein, combined with content determination of the known active components, a comparative experiment of two doses was designed in this study to enhance the reliability of network pharmacology and further analyze the mechanisms of THF in AIS. The framework of this study is shown in Figure 1.

## Materials and Methods

### Materials and Reagents

LC-20AT HPLC (Shimadzu Corporation, Japan), Welch Ultimate XB-C18 column (250 mm × 4.6 mm, 5 μm), methanol and acetonitrile (chromatographic purity, Thermo Fisher Scientific, Shanghai, China), ultrapure water (Hangzhou Wahaha Beverage Co., Ltd., Hangzhou, China), ethanol, and other reagents were used as analytical reagents. LQ (batch number 140610003, purchased from Beijing Qiancao Chinese Medicine Tablet Co.), DNX (batch number 409051, purchased from Sinopharm Group Beijing Huamiao Pharmaceutical Co. Ltd.). HL (batch number 1402001), DZY (batch number 1406001), and DH (batch number 1402001) were purchased from Anguo Changda Chinese herbal medicine Pieces Co. Ltd. (Baoding, China). The identity of all the herbs was confirmed by a botanical expert, Professor Hui Li (Institute of Chinese Materia Medica, China Academy of Chinese Medical Sciences, Beijing, China), and the specimens were archived in the institute. Berberine hydrochloride (lot:110713-201212, purity: 86.7%) and phillyrin (lot:110821-201213, purity: 95.3%), obtained from the National Institutes for Food and Drug Control (Beijing, China), were used as reference standards.

### Preparation of THF

The composition of THF was HL (9 g), DH (5 g), LQ (10 g), DZY (9 g), and DNX (9 g). LQ (166.7 g) and DH (83.3 g), ratio: 2:1, were extracted three times by reflux extraction with 65% ethanol (eight times the volume) for 2 h at a time and filtered and concentrated into a filtrate with a relative density of 1.10–1.20 (50–60°C). HL (150 g), DNX (150 g, wrap-boiling), and DZY (150 g), ratio: 1:1:1, were extracted with water three times (2 h each time): the first time with 10 times the volume of water and the second and third times with eight times the volume of water. The extracts were filtered and concentrated into a filtrate with a relative density of 1.20–1.30 (60–70°C). The two filtrates were mixed, dried under reduced pressure, and finally crushed into a powder.

### Sample and Reference Solution Preparation

Preparation of standard solutions: berberine hydrochloride and phillyrin were prepared in methanol to yield 0.15 and 0.20 mg/ml control solution, respectively, and then filtered through a 0.45-μm membrane filter before HPLC analysis. To prepare a sample solution for berberine, epiberberine, coptisine, and palmatine: THF dried powder (0.5 g) was added to 50 ml of methanol-hydrochloric acid (100: 1) and then sonicated for 30 min. Methanol was added to restore the solution weight to the initial. After filtration, 2 ml of the filtrate was placed in a 10-ml volumetric flask and methanol was added till it reached the mark. The sample solution was obtained by filtration through a 0.45-μm membrane. To prepare a sample solution for phillyrin, 1.0 g of THF dried powder was weighed and 15 ml 70% methanol was added, follow by sonication for 30 min, 70% methanol was used to replace the lost weight, and ultimately the sample solution of phillyrin was obtained by filtration through a 0.45-µm membrane.

### HPLC Analysis

The concentrations of THF compounds were analyzed by HPLC. HPLC analysis was performed using a Welch Ultimate XB-C18 column (250 mm × 4.6 mm, 5 μm).

HPLC conditions for berberine, epiberberine, coptisine, and palmatine were as follows: octadecylsilane-bonded silica gel was used as the filler for the HPLC column. Acetonitrile (0.05 mol/L): potassium dihydrogen phosphate (50:50) ( 0.4 g sodium dodecyl sulfate per 100 ml is added and then the pH is adjusted to 4.0, with phosphoric acid) were used as the mobile phase. Chromatographic analysis was performed at 30°C with a flow rate of 1 ml/min, detection wavelength of 345 nm, and an injection volume of 10 µl.

To perform HPLC for phillyrin, the mobile phase consisted of water (A) and acetonitrile (B), and the gradient elution program was (time/B%): 0–30 min, 23–24%; 30–31 min, 24–95%; 31–39 min, 95%; 39–40 min, 95–23%; 10–15 min, 50–80%; and 40–50 min, 23%. Chromatographic analysis was performed at 30°C with a flow rate of 1 ml/min, detection wavelength of 277 nm, and an injection volume of 10 µl.

### Screening Conditions for Candidate Active Ingredients

The Traditional Chinese Medicine Database and Analysis Platform (TCMSP) was used to screen the active ingredients of THF. The TCMSP is a recognized Chinese herbal medicine platform that provides detailed pharmacokinetic data of herbal ingredients, such as oral bioavailability (OB), drug-likeness (DL), aqueous solubility, and the blood–brain barrier (BBB) *etc*.(Veber et al., 2002; Ru et al., 2014). Due to the breakdown of the neurovascular unit and BBB after cerebral infarction, huge amounts of herbal components directly enter the CNS without obstacles. Thus, the screening conditions on the platform were set as OB ≥30% and DL ≥0.18 (Huang et al., 2014).

### Candidate Active Compounds and Ingredient-Related Target Prediction

THF compounds were clarified from the TCMSP (https://old.tcmsp-e.com/tcmsp.php), ETCM (http://www.tcmip.cn/ETCM/index.php/Home/Index/), TCMID (http://www.megabionet.org/tcmid/), and BATMAN (http://bionet.ncpsb.org/batman-tcm/index.php) databases and literature search. Subsequently, the TCMSP platform was used to identify target genes. In addition, the targets were predicted using the Swiss Target Prediction database (http://www.swisstargetprediction.ch/) for the quality marker (Q-maker) of each herb in THF. Subsequently, different ID types of the targets were transformed into UniProt IDs using the UniProt database (https://www.uniprot.org/).

### Screening of Disease-Associated Targets

AIS-related targets were predicted using GeneCards (https://www.genecards.org) and OMIM (https://omim.org). “Cerebral infarction,” “stroke,” and “cerebral ischemia” were used as the keywords for target searching. The relevance score was set as greater than or equal to 5, duplicate targets were excluded, and cerebral infarction–related targets were screened out. Intersection of THF-AIS–related target genes was performed to verify common target genes.

### Protein–Protein Interaction (PPI) and Component–Target Network

Common targets of THF and AIS were uploaded into the STRING database (https://string-db.org/) for PPI network mapping by setting species as “Homo sapiens” and moderate confidence. Subsequently, the PPI network and component–target network were created and visualized using Cytoscape 3.8.0 software, and the degree centrality, betweenness centrality, and closeness centrality were calculated. The size and color of a node represent the degree value, which further reflects its importance and the number of connections with other nodes.

### Enrichment Analysis

The ClusterProfiler software package and R software were used to perform Gene Ontology (GO) function and Kyoto Encyclopedia of Genes and Genomes (KEGG) pathway enrichment analyses. GO function enrichment analysis involves biological processes (BP), molecular function (MF), and cellular component (CC) biology.

### Molecular Docking

The target receptors were retrieved by downloading them from the PDB database (PDB: https://www.rcsb.org/). The compounds were downloaded from PubChem (https://pubchem.ncbi.nlm.nih.gov/). Prior to docking, all receptor proteins were treated with PyMol 2.5, including the removal of water molecules, salt ions, and small molecules. Molecular docking was then accomplished using AutoDock Vina 1.1.2 software, and docking was performed using the default docking parameters. The docking results were visualized using PyMol 2.5.

### MCAO Models

All experimental procedures complied with the national guidelines for care and the use of laboratory animals were approved by the Experimental Animal Ethics Committee of Capital Medical University (Ethics Number: aeei-2019-086). All male 8-week-old Sprague–Dawley rats were obtained from Beijing Vital River Laboratory Animal Technology Co., Ltd. (License Number: scxk[Beijing]2016-0006) and raised in the experimental animal center of Capital Medical University in a specific pathogen-free SPF environment (18–22°C, 40–60% humidity, 12 h day/night). Twelve male rats were used to avoid the protective effects of estrogen. The rats were anesthetized using 5% isoflurane (Beyotime, China), and the right common carotid artery (CCA), internal carotid artery (ICA), and external carotid artery (ECA) were gently dissected and exposed. After permanent ligation of the CCA and ECA in the proximal portion, the distal end of the ICA was clamped using hemostatic forceps, and a small incision was made at the bifurcation of the CCA. The preprocessed nylon suture was then inserted from the CCA into the ICA for 18–22 mm until resistance was probed in the middle cerebral artery. Finally, a nylon suture (Beijing Shadong Biotechnology Co., Ltd., Beijing, China) was fixed to prevent spontaneous withdrawal, and the incision was sutured and disinfected. The sham group was treated using the same procedure but without MCAO.

### Drug Treatment

The THF dried powder (purity: 5.34 g crude drug/kg dried powder; batch number: 201809-jgf) was provided by the China Academy of Chinese Medical Sciences. The daily consumption of THF is 4.9 g crude drug/kg in rats, which is equivalent to 0.7 g crude drug/kg in patients. We set low dose of THF (THF-L) and high dose of THF (THF-H) groups in rats, approximately one and two times the dosage of clinical patients, respectively: 0.92 g dried powder/kg; 1.84 g dried powder/kg.

### Grouping

The animals were randomly assigned to four groups: sham group, model group (MCAO rats), THF-L group (0.92 g dried powder/kg), and THF-H group (1.84 g dried powder/kg). For the THF-L group, 9.2 g of dry powder was weighed and dissolved in 100 ml of deionized water, meaning 10 ml of the solution contained 0.92 g of the drug, and the volume administered to rats was 10 ml/kg/d. For the THF-H group, 18.4 g of dry powder was weighed and dissolved in 100 ml of deionized water, meaning 10 ml of the solution contained 1.84 g of the drug, and the volume administered to rats was 10 ml/kg/d. The rats in the administration group were intragastrically administered 6, 24, 48, and 72 h after cerebral ischemia surgery. The rats in the sham and model groups were administered the same volume of saline (10 ml/kg/day) at the same time points.

### Immunofluorescence

For immunofluorescence (Shah et al., 2017), the rats in each group were euthanized, rinsed with warm saline *via* rapid left ventricular perfusion, and fixed with 4% paraformaldehyde in PBS solution for 60 min. This process was carried out at 3 days and 30 min post injury (dpi) for immunofluorescence studies. The striatal level of the brain was then dissected and fixed with 4% paraformaldehyde in PBS solution for 1 week. After that, the tissues were dehydrated with gradient ethanol, made transparent with xylene, embedded in paraffin, and cut into 0.5-μm sections using a biological tissue microtome. After dewaxing and hydration, the slides were immersed in 5% BSA for 1 h to block nonspecific antibodies. All paraffin sections were then incubated with primary antibodies overnight at 4°C, namely, GFAP (1:1000, rabbit source, Servicebio, Wuhan, China) and C3(1:400, mouse source, Santa Cruz, United States). These sections were then incubated with Alexa Fluor 594 and Alexa Fluor 488 (1:300, goat anti-mouse and goat anti-rabbit source, Abcam, United Kingdom) for 2 h.

### Immunohistochemistry

For immunohistochemistry (Kusakawa et al., 2015), the slides were incubated with antibodies against caspase-3 (1:750, rabbit source, Servicebio, Wuhan, China), TNF-α (1:500, rabbit source, Servicebio, Wuhan, China), and IL-6 (1:1000, rabbit source, Servicebio, Wuhan, China) overnight at 4°C. Subsequently, paraffin sections of the tissues were washed with PBS, and the secondary antibody was added and incubated for 1 h.

### TUNEL Staining

TUNEL staining (Beyotime Biotechnology, Shanghai, China) was used to detect apoptosis in tissues (Lv et al., 2021). First, the slides were fixed with 4% paraformaldehyde for 30 min at room temperature. The tissue sections were then treated with permeabilization solution for 5 min, followed by TUNEL solution for 1 h at 37°C. Finally, the tissues were observed under a fluorescence microscope (Nikon Eclipse E600, Boston, United States).

### Statistical Analysis

Statistical analyses were performed using SPSS 26.0 (IBM Corp., Armonk, NY, United States). All data are expressed as the mean ± standard deviation. Homogeneity of variance was tested; one-way analysis of variance was used to compare multiple groups; and subsequently, the LSD test was performed for pairwise comparison. *P* < 0.05 was considered to be statistically significant.

## Results

### Quantitative Analysis of Key Compounds

**FIGURE 2:**
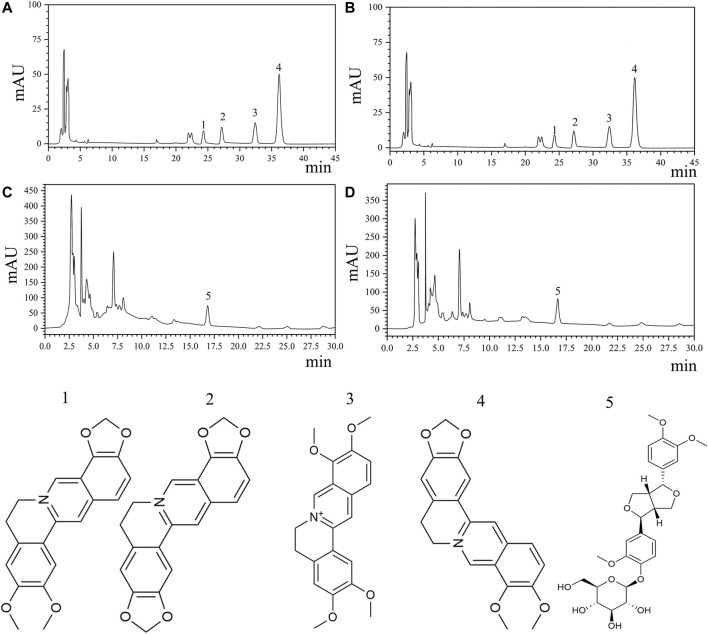
Quantitative analysis of key compounds of THF by HPLC analysis. **(A**,**B)** HPLC analysis and chemical structures of four major ingredients of HL. 1. Epiberberine (C_20_H_17_NO_4_). 2. Coptisine(C_19_H_13_NO_4_). 3. Palmatine (C_21_H_21_NO_4_). 4. Berberine(C_20_H_17_NO_4_). **(C**,**D)** HPLC analysis and chemical structure of phillyrin. 5. Phillyrin(C_20_H_34_O_11_).

HL is a monarch medicine, and LQ has the highest dosage in THF. A qualitative study of HL and LQ is important for controlling the quality of the entire prescription. Therefore, berberine, epiberberine, coptisine, palmatine of HL, and phillyrin of LQ were selected as quality control indices according to the standards of the Chinese Pharmacopoeia 2015 edition. HPLC was used to determine the concentrations of the key compounds. The results are shown in Figure 2 and Table 1. The average berberine, epiberberine, coptisine, palmatine, and phillyrin contents were 34.455, 4.266, 7.205, 9.291, and 4.22 mg/g, respectively.

**TABLE 1:** Content of key compounds of THF.

Ingredients	Content(mg/g)A	Content(mg/g)B	Mean
Epiberberine	4.3425	4.1895	4.266
Coptisine	7.3395	7.0716	7.205
Palmatine	9.438	9.1455	9.291
Berberine	35.1382	33.7735	34.455
Total alkaloids	56.26	54.18	55.22
	Content(mg/g)C	Content(mg/g)D	
Phillyrin	4.16	4.27	4.22

### Candidate Active Compound of THF

Traditional Chinese medicine is a multitarget, multicomponent therapy model. By screening the TCMSP, ETCM, TCMID, and BATMAN databases, a total of 64 active ingredients were retrieved, of which 42 had regulatory effects on cerebral infarction. DH, HL, LQ, DNX, and DZY contained eight, 10, 16, five, and one chemical compounds/compound, respectively. Beta-sitosterol is a common component of DH and DNX, whereas quercetin is a common component of HL and LQ (Supplementary Table S1).

### Common Targets of THF and AIS

**FIGURE 3:**
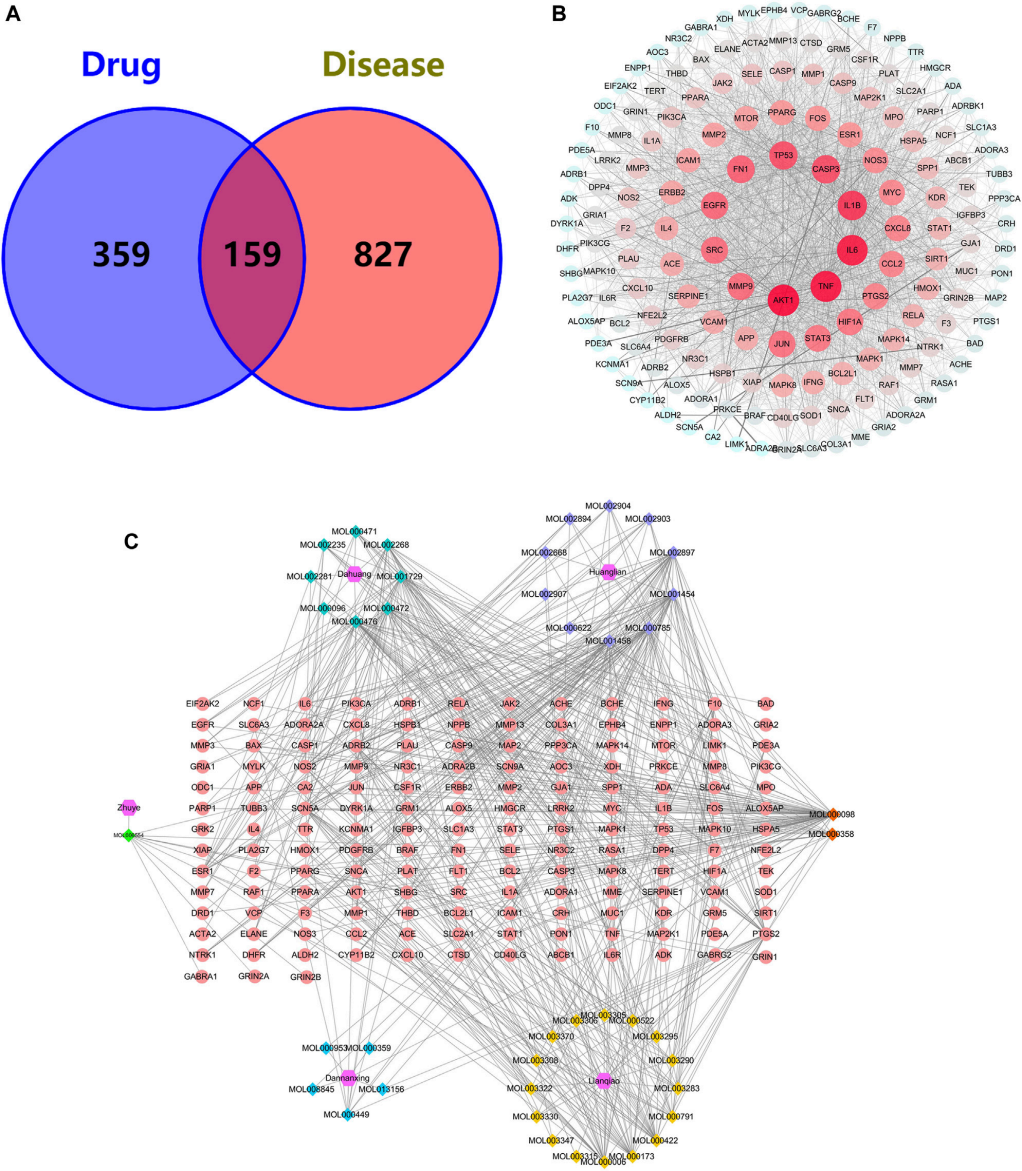
**(A)** Venn diagram for common genes of THF and AIS. **(B)** PPI network of the potential targets of THF. **(C)** Compound–target network of THF on AIS. The regular hexagon, rhombus, and ellipse represent the herbs, herb compounds, and genes, respectively. The number of connected lines indicates the importance of the node.

A total of 518 targets were obtained by eliminating duplicate targets (Supplementary Table S2). These drug targets were further converted into Uniport ID. A total of 986 AIS-related genes were obtained from the GeneCards and OMIM databases. Finally, 159 common target genes of THF and AIS were identified (Figure 3A).

### Protein–Protein Interaction Network

To identify the predominant common targets of THF and AIS, a network analysis of PPI was performed using the STRING database (Figure 3B). A total of 159 common target genes were imported into the STRING database platform, and the PPI network was constructed using Cytoscape 3.8.0 software. The network included 159 nodes and 2,779 edges, with an average degree value of 34.95 (Supplementary Table S3). The larger the node, the more important the role it plays in the network, and the top five targets contain AKT1, TNF, IL6, IL1B, and CASP3 (Table 2), which are not only important genes of THF for treating AIS but are also essential targets for experimental verification.

**TABLE 2:** Topological parameters of the targets.

Name	Degree	Betweenness centrality	Closeness centrality
AKT1	115	0.08234	0.782178
TNF	112	0.059641	0.76699
IL6	110	0.056531	0.763285
IL1B	101	0.033635	0.731481
CASP3	94	0.035664	0.705357
TP53	93	0.022581	0.702222
FN1	88	0.023245	0.686957
EGFR	86	0.022094	0.681034
SRC	84	0.023343	0.67234
MMP9	84	0.018505	0.666667
JUN	83	0.017286	0.669492
STAT3	81	0.013424	0.661088
HIF1A	81	0.011461	0.666667
PTGS2	77	0.010596	0.650206
CCL2	77	0.009268	0.647541
CXCL8	77	0.011757	0.647541
MYC	75	0.014225	0.644898
NOS3	74	0.033877	0.650206
ESR1	71	0.01457	0.639676
FOS	71	0.020903	0.639676

### Compound-Target Network Analysis

To establish the correlation between THF and potential targets, the “Compound–Target Network” was conducted using Cytoscape 3.8.0 (Figure 3C). This network includes 206 nodes and 604 edges, among which the regular hexagon, rhombus, and ellipse represent herbs, herb compounds, and genes, respectively. The degree values, which reflect the number of nodes directly linked to the compound, were used to evaluate the importance of the compound in the network. We selected the top 15 compounds based on their degree values, as shown in Table 3. The results showed that quercetin (MOL000098, degree = 70), coptisine (MOL001458, degree = 38), palmatine (MOL000785, degree = 38), berberine (MOL001454, degree = 36), epiberberine (MOL002897, degree = 36), physcion (MOL000476, degree = 35), emodin (MOL000472, degree = 30), and chrysophanol (MOL001729, degree = 29) were identified. These compounds exhibited high degree values, demonstrating that one herb can target multiple genes and play a crucial role in the treatment of cerebral infarction.

**TABLE 3:** Core compounds of THF.

Name	Compound	Degree	Betweenness	Closeness
MOL000098	Quercetin	70	13738.66	0.465909
MOL001458	Coptisine	38	4416.309	0.403543
MOL000785	Palmatine	38	4259.88	0.403543
MOL001454	Berberine	36	4012.556	0.406746
MOL000476	Physcion	36	3922.645	0.395753
MOL002897	Epiberberine	35	3346.933	0.405138
MOL000472	Emodin	30	3200.005	0.386792
MOL001729	Crysophanol	29	2903.895	0.382463
MOL000006	Luteolin	26	2134.274	0.372727
MOL000173	Wogonin	25	1742.017	0.382463
MOL000422	Kaempferol	24	1383.854	0.370036
MOL002268	Rhein	22	1666.857	0.375458
MOL000358	Beta-sitosterol	17	1651.767	0.364769
MOL000791	Bicuculline	14	1259.112	0.357143

### GO and KEGG Pathway Enrichment Analysis

**FIGURE 4:**
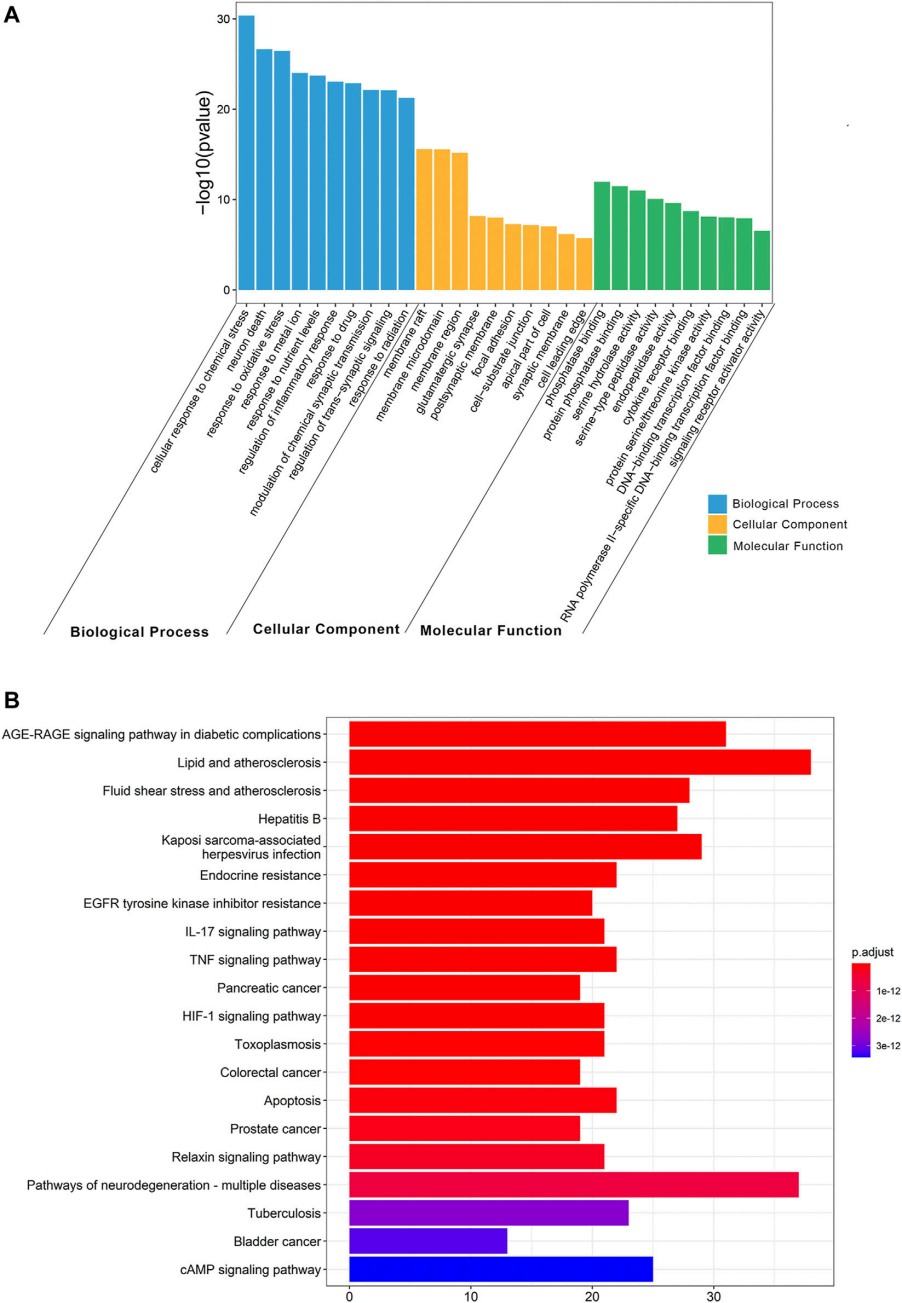
Gene Ontology (GO) and Kyoto Encyclopedia of Genes and Genomes (KEGG) pathway enrichment analysis. **(A)** GO functional enrichment analysis. **(B)** KEGG pathway enrichment analysis.

To determine the biological characteristics of potential targets of THF on AIS, GO and KEGG pathway enrichment analyses on 159 common targets were performed using the ClusterProfiler software package of the R platform. A total of 2681 GO functional processes were obtained and annotated in terms of BP, MF, and CC (*p* <0.05). The top 10 significant terms in the BP, CC, and MF categories are shown in Figure 4A. Among them, the top three enriched GO functions for target genes included cellular response to chemical stress, neuron death, response to oxidative stress in BP, membrane rafts, membrane microdomains, membrane regions in CC, phosphatase binding, protein phosphatase binding, and serine hydrolase activity in MF. KEGG pathway enrichment analysis showed that 168 pathways were associated with cerebral infarction. Detailed pathways related to cerebral infarction are shown in Figure 4B and Supplementary Table S4. Enrichment analysis suggested that the inflammation and apoptotic pathways were enriched by multiple targets. Collectively, these results suggest that restraining inflammation and apoptosis may be a promising therapeutic strategy for AIS treated with THF.

### Molecular Docking

**FIGURE 5:**
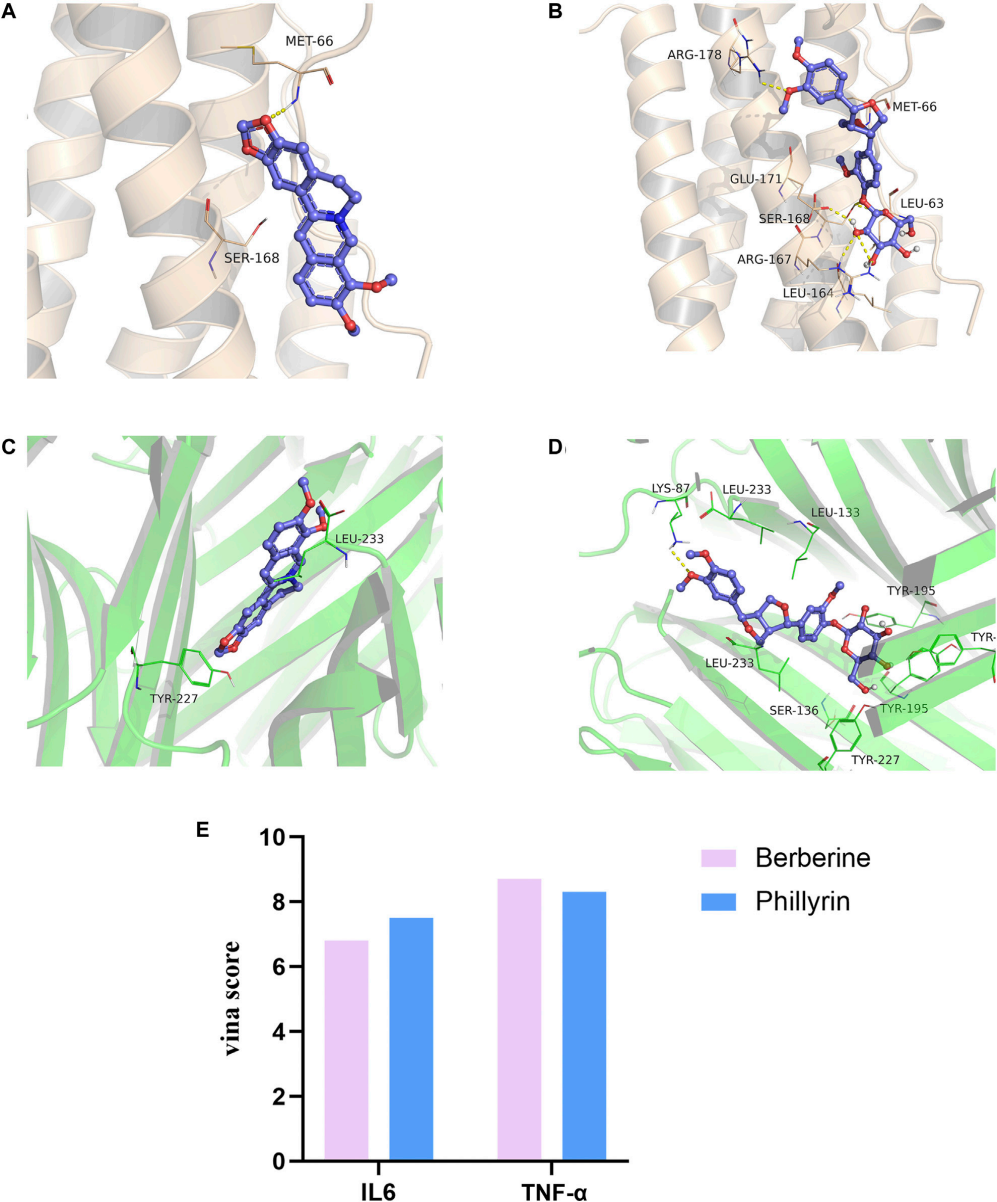
Molecular docking. **(A)** Berberine-(IL-6). **(B)** Phillyrin-(IL-6). **(C)** Berberine-(TNF-α). **(D)** Phillyrin-(TNF-α). **(E)** Absolute value of vina score (kcal/mol).

Berberine (the most abundant component in HL) and phillyrin were used for molecular docking. The key targets IL-6 and TNF-α were also selected for analysis. It is generally accepted that the lower the Vina score, the more stable the binding of the compound is to the crucial target. The results indicated that berberine and phillyrin had good binding activity for IL-6 and TNF-α (Figures 5A–E).

### Inhibition of THF Activity in Apoptosis in Cerebral Infarction in the MCAO Model

**FIGURE 6:**
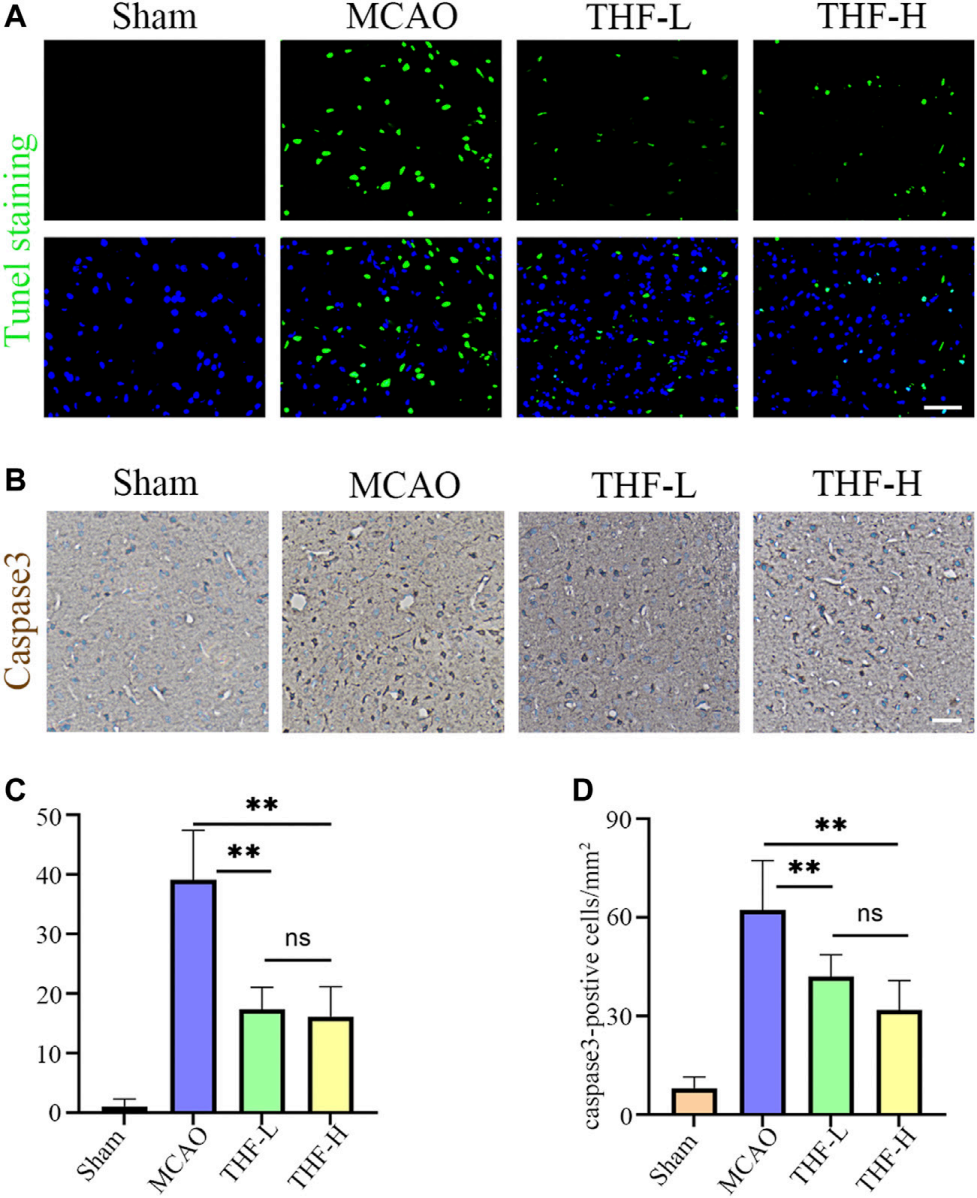
Effects of THF on the neural cell apoptosis in infarction focus at 3 days and 30 min. **(A)** TUNEL staining of apoptotic cells in the cortex. Scale bar = 50 μm. **(B)** Representative immunohistochemistry images showing caspase-3–positive cells in the cortex. Scale bar = 100 μm. **(C)** Quantitative analysis of apoptotic cells in the cortex. **(D)** Quantification of the number of caspase-3–positive cells in each group. *n* = 3. (^**^ *p* < 0.01, compared to the MCAO group).

As network pharmacology analysis suggested that THF may protect against neuronal damage by inhibiting the apoptotic pathway, TUNEL staining was used to estimate the number of apoptotic cells at the infarction locus. A marked increase in apoptotic cells was observed in the cortex of MCAO rats, whereas THF-L and THF-H remarkably reduced apoptosis (Figures 6A,C). Immunochemistry was further performed to determine the protective effect of THF on neural cells. The number of caspase-3–positive cells in the cortex was significantly lower than that in the MCAO group. In contrast to MCAO rats, the number of cells with positive expression of caspase-3 in the THF-L and THF-H groups was significantly decreased (Figures 6B,D). These results indicate that THF treatment significantly suppressed the expression of caspase-3 and protected neural cells from apoptosis in MCAO rats.

### Inhibitory Activity of THF on the Pro-inflammatory Activation of A1 Astrocyte and Release of IL-6 and TNF-α

**FIGURE 7:**
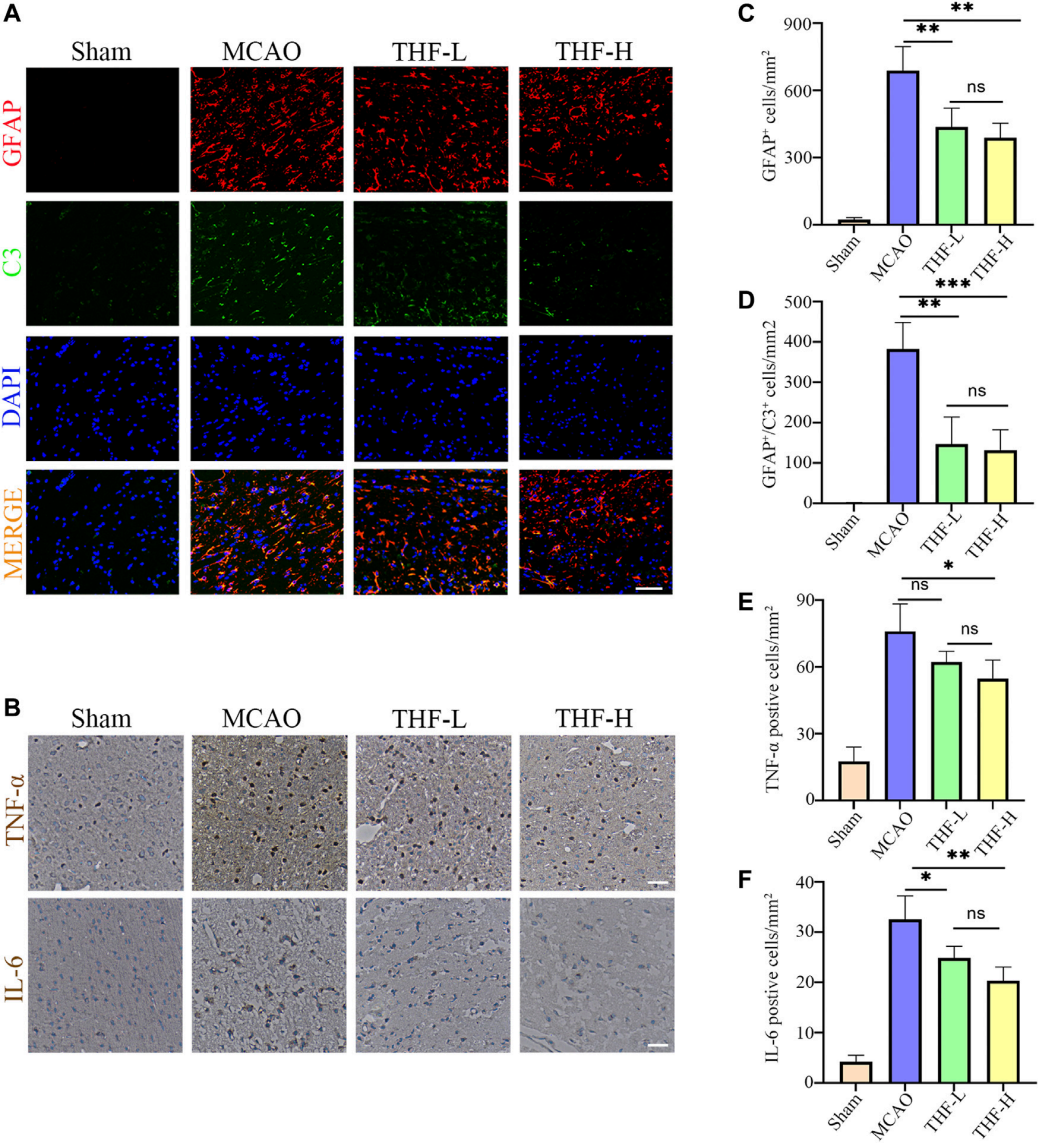
THF inhibits the activation of A1 astrocytes and expression of IL-6 and TNF-α. **(A)** Representative immunofluorescence images represent GFAP (red) and C3 (green) staining in the cortex. GFAP, glial fibrillary acidic protein. Scale bar = 50 μm. **(B)** Immunohistochemistry of IL-6 and TNF-α in the cortex. Scale bar = 100 μm. **(C)** Quantification of the number of GFAP positive cells in each group. **(D)** Statistical diagram of GFAP/C3 positive cells. **(E)** Statistical diagram of TNF-α–positive cells. **(F)** Quantification of the density of IL-6–immunostained cells. IL-6, interleukin-6; TNF-α, tumor necrosis factor-α. *n* = 3. (^*^ *p* < 0.05, ^**^ *p* < 0.01, ^***^ *p* < 0.001compared to the MCAO group).

In the network pharmacology analysis, IL-6 and TNF were the hub genes of the PPI network, and the TNF signaling pathway was the key pathway in the KEGG enrichment, suggesting that the inflammatory response may play a pivotal role in treating AIS by THF. To evaluate the inflammatory response after cerebral infarction, immunofluorescence was performed to determine the activation of A1 astrocytes that induce an evident inflammatory response in many CNS diseases. First, double immunofluorescence staining of GFAP and C3 was performed to label astrocytes, in which positive cells labeled with C3/GFAP represented A1-type pro-inflammatory astrocytes, whereas positive cells labeled with GFAP represented all types of astrocytes. In this study, a large number of C3-/GFAP-labeled positive cells were detected at the edge of the infarct foci in MCAO rats, but not in the ipsilateral brains of rats in the sham group. Furthermore, the statistical results showed that the cell density of GFAP and C3/GFAP positive cells around infarct lesions in the THF-L and THF-H groups was lower than that in MCAO rats (Figures 7A,C,D). Simultaneously, immunohistochemical staining was performed to evaluate the release of IL-6 and TNF-α. Significant differences were observed between the sham and model groups. Accordingly, IL-6 expression in the cortex was decreased in both the THF-L and THF-H groups compared with that in the MCAO group, whereas THF-H markedly reduced TNF-α expression, suggesting that THF inhibits the release of inflammatory factors (Figures 7B,E,F). Collectively, our study demonstrated that THF curbs the inflammatory response and thereby protects neural cells from apoptosis during cerebral infarction.

## Discussion

AIS is a common disease in elderly people, with a stepwise sequence of pathophysiological processes that are strongly correlated to inflammatory responses and apoptosis during development and progression (Ao et al., 2018; Uzdensky, 2019). THF is a traditional herbal medicine formula with multiple active compounds, which has been used in treating AIS for more than 10 years in China owing to its excellent efficacy in “heating–clearing, fire–purgingg, and detoxification.” In this study, network pharmacology and experimental verification were applied to explore the underlying mechanism by which THF improves AIS outcomes.

Network pharmacological analysis was first used to predict and determine the active ingredients and potential targets of THF in AIS. First, 42 potential compounds associated with the therapeutic effects of cerebral infarction were identified. By constructing a PPI network for the common targets of THF and AIS, AKT1, TNF, IL6, IL1B, and CASP3 were predicted as pivotal targets. Finally, KEGG pathway enrichment analysis showed that inflammation- and apoptosis-related pathways were significantly enriched, and we focused on the TNF pathway. IL-6 is a pleiotropic cytokine primarily produced by activated microglia and astrocytes. Upregulation of IL-6 may act on microvascular endothelial cells and increase the expression of adhesion molecules and chemokines, thus mediating the inflammatory cascade and aggravating cerebral ischemic injury (Huang et al., 2006). CASP3 is generally recognized as a crucial molecule in apoptosis after cerebral ischemia. Thus, inhibition of the CASP3 pathway can simultaneously block neural cell apoptosis (Burguillos et al., 2011). TNF-α (tumor necrosis factor–α) is a cytokine associated with systemic inflammation, immune regulation, inhibition of tumor cell growth, and rejection of organ transplantation. There is considerable evidence that TNF-α is significantly associated with ischemic stroke, which further increases sharply in the acute stage of stroke, resulting in serious tissue damage (Shishkova et al., 2018; Zuo et al., 2019). Based on the HPLC analysis and molecular docking results, we speculated that berberine and phillyrin are likely to play a pivotal role in inflammation. Previous studies have shown that berberine alleviates cerebral ischemic injury by attenuating apoptosis, inhibiting M1 glial cell polarization, and inhibiting the expression of pro-inflammatory cytokines (Simões Pires et al., 2014; Zhu et al., 2019). Similarly, *in vivo* experiments showed that phillyrin inhibits microglia-mediated inflammation by promoting the polarization of “M2” microglia. In addition, phillyrin has been reported to alleviate apoptosis by inhibiting apoptotic pathways such as caspase-3 (Du et al., 2020; Jiang et al., 2021). Altogether, the network pharmacology results showed that TNF, IL6, and CASP3 are hub genes in the PPI network. In addition, KEGG enrichment analysis showed that the TNF inflammatory pathway was significantly enriched through multiple targets (including the hub targets CASP3 and IL6). These targets are actively involved in apoptotic and inflammatory pathways, suggesting that THF may exert anti-inflammatory and antiapoptotic effects on AIS by primarily regulating the TNF signaling pathway and targeting certain genes, such as CASP-3, IL-6, and TNF-α. Therefore, corresponding animal experiments were subsequently designed to validate the predictions of network pharmacology. Specifically, we emphasize that the CASP3 signaling pathway induces apoptosis and detects the activation of A1 astrocytes and the release of IL6 and TNF-α. CASP3, a marker of apoptosis, is located in the core of the apoptotic cascade pathway. Nerve cells are very sensitive to severe ischemia and hypoxia and die rapidly after ischemic stroke. In particular, the number of CASP3-positive neurons in the cerebral cortex and hippocampal CA1 area increased at 3 h, reached a peak at 48 h, and decreased to a stable level 14 days after MCAO (Liu et al., 2013). Therefore, inhibiting the expression of CASP3 and reducing neural cell apoptosis are important therapeutic targets for acute ischemic stroke (Akpan and Troy, 2013). Our findings confirm that THF alleviates apoptosis of neural cells by inhibiting the CASP3 pathway.

Numerous studies have demonstrated that abnormally activated A1 astrocytes exhibit a detrimental effect on the transmission of inflammatory mediators and glutamic acids, initiating immune cascade reactions and leading to increased tissue damage, such as the destruction of the BBB, brain edema, and neuronal degeneration and death (Hawkins and Davis, 2005; Buffo et al., 2010). TNF-α is an important mediator of the inflammatory response, which can activate microglia and astrocytes, leading to leakage of the BBB and aggravating the progression of stroke (Berti et al., 2002; Tuttolomondo et al., 2014). IL-6 is a pro-inflammatory cytokine that promotes the occurrence and development of neurovascular unit dysfunction and neuroinflammation (Pawluk et al., 2020). Therefore, restraining the abnormal activation of A1 astrocytes and production of TNF-α and IL-6 is conducive to alleviating neuronal damage after cerebral ischemia, which is also indispensable for functional rehabilitation. In our study, A1 astrocytes were significantly activated, and the levels of TNF-α and IL-6 were upregulated in MCAO rats, consequently causing neuronal death. However, this inflammation and tissue injury could be reversed by THF treatment. These data suggest a potential mechanism whereby THF can curb overactivation of A1 astrocytes and the release of TNF-α and IL-6 at an early stage after AIS.

Finally, although our study demonstrates that THF has an impact on the inflammatory response and apoptosis by targeting IL-6, TNF-α, and caspase-3 in MCAO rats, the upstream pathway has not been elucidated. In addition, a previous study showed that THF can improve clinical outcomes by altering the gut microbiota (Guo Q. et al., 2021). However, there is currently no evidence of the efficacy of THF in animals. Therefore, the effect of THF on improving animal prognosis and more in-depth mechanisms remain to be elucidated in the future.

## Conclusion

To the best of our knowledge, ischemic stroke is a complex pathological process mediated by multiple targets and multiple pathways, and the complicated pathophysiological mechanism of THF in treating AIS was explored based on network pharmacology analysis and experimental validation. According to network pharmacology analysis, 42 bioactive compounds and 159 targets of THF related to AIS were obtained. Simultaneously, AKT1, TNF, IL6, IL1B, and CASP3 were identified as hub targets. Inflammation and apoptosis were significantly enriched in multiple signaling pathways and multiple targets. Therefore, THF is likely to be involved in modulating AIS by primarily regulating the TNF signaling pathway and targeting certain genes such as CASP3, IL-6, and TNF-α. Based on experimental validation, this study provides new insights into the mechanisms by which THF can effectively decrease neural cell apoptosis through the caspase-3 pathway and restrain the excessive activation of A1 astrocytes. Therefore, THF may serve as a prospective treatment strategy for AIS through multiple targets, components, and pathways.

## Data Availability

The original contributions presented in the study are included in the article/Supplementary Material, further inquiries can be directed to the corresponding authors.
