# Supplementary material for: Tanhuo Formula Inhibits Astrocyte Activation and Apoptosis in Acute Ischemic Stroke
Source: Front Pharmacol. 2022 Apr 26;13:859244. doi: 10.3389/fphar.2022.859244 (PMC9087855; doi:10.3389/fphar.2022.859244)
Supplement: Supplementary file 1 [file Table1.DOCX]

**Supplementary Table S1:**Active compounds information of 5 herbs in THF

| Mol ID | Compound | OB(%) | DL | Medicine |
| --- | --- | --- | --- | --- |
| MOL002235 | EUPATIN | 50.80308 | 0.40804 | DaHuang |
| MOL002251 | Mutatochrome | 48.63848 | 0.61196 | DaHuang |
| MOL002259 | Physciondiglucoside | 41.64856 | 0.63145 | DaHuang |
| MOL002260 | Procyanidin B-5,3'-O-gallate | 31.99116 | 0.32148 | DaHuang |
| MOL002268 | Rhein | 47.06521 | 0.27678 | DaHuang |
| MOL002276 | Sennoside E_qt | 50.68506 | 0.61108 | DaHuang |
| MOL002280 | Torachrysone-8-O-beta-D-(6'-oxayl)-glucoside | 43.01996 | 0.73687 | DaHuang |
| MOL002281 | Toralactone | 46.46436 | 0.23965 | DaHuang |
| MOL002288 | Emodin-1-O-beta-D-glucopyranoside | 44.80639 | 0.79742 | DaHuang |
| MOL002293 | Sennoside D_qt | 61.05623 | 0.61109 | DaHuang |
| MOL002297 | Daucosterol_qt | 35.88889 | 0.70418 | DaHuang |
| MOL002303 | palmidin A | 32.4504 | 0.65 | DaHuang |
| MOL000358 | beta-sitosterol | 36.91391 | 0.75123 | DaHuang |
| MOL000471 | aloe-emodin | 83.37964 | 0.2409 | DaHuang |
| MOL000554 | gallic acid-3-O-(6'-O-galloyl)-glucoside | 30.25032 | 0.6746 | DaHuang |
| MOL001729 | Crysophanol | 18.64 | 0.21 | DaHuang |
| MOL000472 | emodin | 24.4 | 0.24 | DaHuang |
| MOL000476 | Physcion | 22.29 | 0.27 | DaHuang |
| MOL000096 | (-)-catechin | 49.67639 | 0.24162 | DaHuang |
| MOL013146 | 8,11,14-Docosatrienoic acid, methyl ester | 43.2255 | 0.30369 | DanNanXing |
| MOL013156 | [(2R)-2-[[[(2R)-2-(benzoylamino)-3-phenylpropanoyl]amino]methyl]-3-phenylpropyl] acetate | 38.88034 | 0.56235 | DanNanXing |
|  |  |  |  |  |
|  |  |  |  | DanNanXing |
| MOL001510 | 24-epicampesterol | 37.57682 | 0.71413 | DanNanXing |
| MOL000358 | beta-sitosterol | 36.91391 | 0.75123 | DanNanXing |
| MOL000359 | sitosterol | 36.91391 | 0.7512 | DanNanXing |
| MOL000449 | Stigmasterol | 43.82985 | 0.75665 | DanNanXing |
| MOL000953 | CLR | 37.8739 | 0.67677 | DanNanXing |
| MOL008845 | Deoxycholic Acid | 40.72 | 0.68 | DanNanXing |
| MOL000622 | Magnograndiolide | 63.70888 | 0.18833 | HuangLian |
| MOL008647 | Moupinamide | 86.71216 | 0.26454 | HuangLian |
| MOL000098 | quercetin | 46.43335 | 0.27525 | HuangLian |
| MOL000785 | Palmatine | 64.60111 | 0.64524 | HuangLian |
| MOL000762 | Palmidin A | 35.35819 | 0.65003 | HuangLian |
| MOL002894 | berberrubine | 35.73551 | 0.7269 | HuangLian |
| MOL013352 | Obacunone | 43.28625 | 0.76724 | HuangLian |
| MOL002903 | (R)-Canadine | 55.36687 | 0.77465 | HuangLian |
| MOL002907 | Corchoroside A_qt | 104.9542 | 0.77599 | HuangLian |
| MOL002897 | Epiberberine | 43.09233 | 0.7761 | HuangLian |
| MOL001454 | Berberine | 36.86125 | 0.77665 | HuangLian |
| MOL002904 | Berlambine | 36.6809 | 0.81596 | HuangLian |
| MOL001458 | Coptisine | 30.67185 | 0.85647 | HuangLian |
| MOL002668 | Worenine | 45.83318 | 0.86552 | HuangLian |
| MOL000173 | wogonin | 30.68457 | 0.22942 | LianQiao |
| MOL000422 | kaempferol | 41.88225 | 0.24066 | LianQiao |
| MOL000006 | luteolin | 36.16263 | 0.24552 | LianQiao |
| MOL000098 | quercetin | 46.43335 | 0.27525 | LianQiao |
| MOL003370 | Onjixanthone I | 79.15712 | 0.29993 | LianQiao |
| MOL003283 | (2R,3R,4S)-4-(4-hydroxy-3-methoxy-phenyl)-7-methoxy-2,3-dimethylol-tetralin-6-ol | 66.51047 | 0.38869 | LianQiao |
|  |  |  |  | LianQiao |
| MOL003290 | (3R,4R)-3,4-bis[(3,4-dimethoxyphenyl)methyl]oxolan-2-one | 52.30461 | 0.4849 |  |
|  |  |  |  | LianQiao |
| MOL003322 | FORSYTHINOL | 81.2475 | 0.56646 | LianQiao |
| MOL003295 | (+)-pinoresinol monomethyl ether | 53.08233 | 0.56703 | LianQiao |
| MOL003306 | ACon1_001697 | 85.11772 | 0.56703 | LianQiao |
| MOL003330 | (-)-Phillygenin | 95.03641 | 0.56703 | LianQiao |
| MOL003308 | (+)-pinoresinol monomethyl ether-4-D-beta-glucoside_qt | 61.20403 | 0.56711 | LianQiao |
|  |  |  |  | LianQiao |
| MOL003347 | hyperforin | 44.03317 | 0.598 | LianQiao |
| MOL003348 | adhyperforin | 44.03419 | 0.61329 | LianQiao |
| MOL003344 | β-amyrin acetate | 42.06007 | 0.74022 | LianQiao |
| MOL000358 | beta-sitosterol | 36.91391 | 0.75123 | LianQiao |
| MOL000211 | Mairin | 55.37707 | 0.7761 | LianQiao |
| MOL003315 | 3beta-Acetyl-20,25-epoxydammarane-24alpha-ol | 33.06511 | 0.79318 | LianQiao |
| MOL003281 | 20(S)-dammar-24-ene-3β,20-diol-3-acetate | 40.23 | 0.82116 | LianQiao |
| MOL000522 | arctiin | 34.44847 | 0.84109 | LianQiao |
| MOL003365 | Lactucasterol | 40.99114 | 0.84862 | LianQiao |
| MOL003305 | PHILLYRIN | 36.39569 | 0.86315 | LianQiao |
| MOL000791 | bicuculline | 69.66746 | 0.88223 | LianQiao |
| MOL006554 | Taraxerol | 38.4 | 0.77 | DanZhuYe |
| MOL003137 | Leucanthoside | 32.12 | 0.78 | DanZhuYe |
