# Supplementary material for: Tanhuo Formula Inhibits Astrocyte Activation and Apoptosis in Acute Ischemic Stroke
Source: Front Pharmacol. 2022 Apr 26;13:859244. doi: 10.3389/fphar.2022.859244 (PMC9087855; doi:10.3389/fphar.2022.859244)
Supplement: Supplementary file 2 [file Table2.DOCX]

**Supplementary Table S2:Target genes of bioactive compounds in THF**

| **Mol ID** | **Compound** | **Gene Name** |
| --- | --- | --- |
| MOL002235 | EUPATIN | NOS2 |
| MOL002235 | EUPATIN | AR |
| MOL002235 | EUPATIN | F10 |
| MOL002235 | EUPATIN | PTGS2 |
| MOL002235 | EUPATIN | F7 |
| MOL002235 | EUPATIN | TOP2A |
| MOL002235 | EUPATIN | ESR2 |
| MOL002235 | EUPATIN | DPP4 |
| MOL002235 | EUPATIN | HSP90AA1 |
| MOL002235 | EUPATIN | PRSS1 |
| MOL002235 | EUPATIN | NCOA2 |
| MOL002235 | EUPATIN | CAMKMT |
| MOL002235 | EUPATIN | F2 |
| MOL002235 | EUPATIN | SCN5A |
| MOL002235 | EUPATIN | KDR |
| MOL002235 | EUPATIN | PPARD |
| MOL002259 | Physciondiglucoside | TOP2A |
| MOL002280 | Torachrysone-8-O-beta-D-(6'-oxayl)-glucoside | TOP2A |
| MOL002281 | Toralactone | NOS2 |
| MOL002281 | Toralactone | PTGS1 |
| MOL002281 | Toralactone | ESR1 |
| MOL002281 | Toralactone | PTGS2 |
| MOL002281 | Toralactone | ESR2 |
| MOL002281 | Toralactone | HSP90AA1 |
| MOL002281 | Toralactone | CHEK1 |
| MOL002281 | Toralactone | PRKACA |
| MOL002288 | Emodin-1-O-beta-D-glucopyranoside | TOP2A |
| MOL002297 | Daucosterol_qt | PGR |
| MOL002297 | Daucosterol_qt | NCOA2 |
| MOL000358 | beta-sitosterol | PGR |
| MOL000358 | beta-sitosterol | NCOA2 |
| MOL000358 | beta-sitosterol | PTGS1 |
| MOL000358 | beta-sitosterol | PTGS2 |
| MOL000358 | beta-sitosterol | HSP90AA1 |
| MOL000358 | beta-sitosterol | KCNH2 |
| MOL000358 | beta-sitosterol | PRKACA |
| MOL000358 | beta-sitosterol | DRD1 |
| MOL000358 | beta-sitosterol | CHRM3 |
| MOL000358 | beta-sitosterol | CHRM1 |
| MOL000358 | beta-sitosterol | SCN5A |
| MOL000358 | beta-sitosterol | CHRM4 |
| MOL000358 | beta-sitosterol | PDE3A |
| MOL000358 | beta-sitosterol | ADRA1A |
| MOL000358 | beta-sitosterol | CHRM2 |
| MOL000358 | beta-sitosterol | ADRA1B |
| MOL000358 | beta-sitosterol | ADRB2 |
| MOL000358 | beta-sitosterol | CHRNA2 |
| MOL000358 | beta-sitosterol | SLC6A4 |
| MOL000358 | beta-sitosterol | OPRM1 |
| MOL000358 | beta-sitosterol | CHRNA7 |
| MOL000358 | beta-sitosterol | BCL2 |
| MOL000358 | beta-sitosterol | BAX |
| MOL000358 | beta-sitosterol | CASP9 |
| MOL000358 | beta-sitosterol | JUN |
| MOL000358 | beta-sitosterol | CASP3 |
| MOL000358 | beta-sitosterol | CASP8 |
| MOL000358 | beta-sitosterol | PRKCA |
| MOL000358 | beta-sitosterol | PON1 |
| MOL000358 | beta-sitosterol | MAP2 |
| MOL000471 | aloe-emodin | PTGS1 |
| MOL000471 | aloe-emodin | PTGS2 |
| MOL000471 | aloe-emodin | HSP90AA1 |
| MOL000471 | aloe-emodin | PRKACA |
| MOL000471 | aloe-emodin | NCOA2 |
| MOL000471 | aloe-emodin | PKIA |
| MOL000471 | aloe-emodin | AKR1B1 |
| MOL000471 | aloe-emodin | IGHG1 |
| MOL000471 | aloe-emodin | CDKN1A |
| MOL000471 | aloe-emodin | EIF6 |
| MOL000471 | aloe-emodin | BAX |
| MOL000471 | aloe-emodin | TNFAIP6 |
| MOL000471 | aloe-emodin | CASP3 |
| MOL000471 | aloe-emodin | TP53 |
| MOL000471 | aloe-emodin | FASN |
| MOL000471 | aloe-emodin | PRKCA |
| MOL000471 | aloe-emodin | PRKCE |
| MOL000471 | aloe-emodin | CDK1 |
| MOL000471 | aloe-emodin | PCNA |
| MOL000471 | aloe-emodin | MYC |
| MOL000471 | aloe-emodin | IL1B |
| MOL000471 | aloe-emodin | PRKCD |
| MOL000471 | aloe-emodin | CCNB1 |
| MOL000471 | aloe-emodin | IL6 |
| MOL000471 | aloe-emodin | TNF |
| MOL000096 | (-)-catechin | PTGS1 |
| MOL000096 | (-)-catechin | ESR1 |
| MOL000096 | (-)-catechin | PTGS2 |
| MOL000096 | (-)-catechin | HSP90AA1 |
| MOL000096 | (-)-catechin | DPEP1 |
| MOL000096 | (-)-catechin | PRKACA |
| MOL000096 | (-)-catechin | NCOA2 |
| MOL000096 | (-)-catechin | CAMKMT |
| MOL000096 | (-)-catechin | FASN |
| MOL000096 | (-)-catechin | PPARG |
| MOL000096 | (-)-catechin | KLF7 |
| MOL013156 | [(2R)-2-[[[(2R)-2-(benzoylamino)-3-phenylpropanoyl]amino]methyl]-3-phenylpropyl] acetate | KCNH2 |
| MOL013156 | [(2R)-2-[[[(2R)-2-(benzoylamino)-3-phenylpropanoyl]amino]methyl]-3-phenylpropyl] acetate | F10 |
| MOL013156 | [(2R)-2-[[[(2R)-2-(benzoylamino)-3-phenylpropanoyl]amino]methyl]-3-phenylpropyl] acetate | PTGS2 |
| MOL001510 | 24-epicampesterol | PGR |
| MOL001510 | 24-epicampesterol | NCOA2 |
| MOL000358 | beta-sitosterol | PGR |
| MOL000358 | beta-sitosterol | NCOA2 |
| MOL000358 | beta-sitosterol | PTGS1 |
| MOL000358 | beta-sitosterol | PTGS2 |
| MOL000358 | beta-sitosterol | HSP90AA1 |
| MOL000358 | beta-sitosterol | KCNH2 |
| MOL000358 | beta-sitosterol | PRKACA |
| MOL000358 | beta-sitosterol | DRD1 |
| MOL000358 | beta-sitosterol | CHRM3 |
| MOL000358 | beta-sitosterol | CHRM1 |
| MOL000358 | beta-sitosterol | SCN5A |
| MOL000358 | beta-sitosterol | CHRM4 |
| MOL000358 | beta-sitosterol | PDE3A |
| MOL000358 | beta-sitosterol | ADRA1A |
| MOL000358 | beta-sitosterol | CHRM2 |
| MOL000358 | beta-sitosterol | ADRA1B |
| MOL000358 | beta-sitosterol | ADRB2 |
| MOL000358 | beta-sitosterol | CHRNA2 |
| MOL000358 | beta-sitosterol | SLC6A4 |
| MOL000358 | beta-sitosterol | OPRM1 |
| MOL000358 | beta-sitosterol | CHRNA7 |
| MOL000358 | beta-sitosterol | BCL2 |
| MOL000358 | beta-sitosterol | BAX |
| MOL000358 | beta-sitosterol | CASP9 |
| MOL000358 | beta-sitosterol | JUN |
| MOL000358 | beta-sitosterol | CASP3 |
| MOL000358 | beta-sitosterol | CASP8 |
| MOL000358 | beta-sitosterol | PRKCA |
| MOL000358 | beta-sitosterol | PON1 |
| MOL000358 | beta-sitosterol | MAP2 |
| MOL000359 | sitosterol | PGR |
| MOL000359 | sitosterol | NCOA2 |
| MOL000359 | sitosterol | NR3C2 |
| MOL000449 | Stigmasterol | PGR |
| MOL000449 | Stigmasterol | NR3C2 |
| MOL000449 | Stigmasterol | NCOA2 |
| MOL000449 | Stigmasterol | NPPB |
| MOL000449 | Stigmasterol | IGHG1 |
| MOL000449 | Stigmasterol | RXRA |
| MOL000449 | Stigmasterol | NCOA1 |
| MOL000449 | Stigmasterol | PTGS1 |
| MOL000449 | Stigmasterol | PTGS2 |
| MOL000449 | Stigmasterol | ADRA2A |
| MOL000449 | Stigmasterol | SLC6A2 |
| MOL000449 | Stigmasterol | SLC6A3 |
| MOL000449 | Stigmasterol | ADRB2 |
| MOL000449 | Stigmasterol | AKR1B1 |
| MOL000449 | Stigmasterol | PLAU |
| MOL000449 | Stigmasterol | LTA4H |
| MOL000449 | Stigmasterol | MAOB |
| MOL000449 | Stigmasterol | MAOA |
| MOL000449 | Stigmasterol | PRKACA |
| MOL000449 | Stigmasterol | CTRB1 |
| MOL000449 | Stigmasterol | CHRM3 |
| MOL000449 | Stigmasterol | CHRM1 |
| MOL000449 | Stigmasterol | ADRB1 |
| MOL000449 | Stigmasterol | SCN5A |
| MOL000449 | Stigmasterol | ADRA1A |
| MOL000449 | Stigmasterol | CHRM2 |
| MOL000449 | Stigmasterol | ADRA1B |
| MOL000449 | Stigmasterol | CHRNA7 |
| MOL000953 | CLR | PGR |
| MOL000953 | CLR | NR3C2 |
| MOL000953 | CLR | NCOA2 |
| MOL008845 | Deoxycholic Acid | PGR |
| MOL008845 | Deoxycholic Acid | NR3C2 |
| MOL008845 | Deoxycholic Acid | NCOA2 |
| MOL008845 | Deoxycholic Acid | NCOA1 |
| MOL002894 | berberrubine | NOS2 |
| MOL002894 | berberrubine | PTGS1 |
| MOL002894 | berberrubine | KCNH2 |
| MOL002894 | berberrubine | ESR1 |
| MOL002894 | berberrubine | AR |
| MOL002894 | berberrubine | SCN5A |
| MOL002894 | berberrubine | PTGS2 |
| MOL002894 | berberrubine | RXRA |
| MOL002894 | berberrubine | PRKACA |
| MOL002894 | berberrubine | PRSS1 |
| MOL002894 | berberrubine | NCOA2 |
| MOL002894 | berberrubine | CAMKMT |
| MOL002903 | (R)-Canadine | PTGS1 |
| MOL002903 | (R)-Canadine | CHRM3 |
| MOL002903 | (R)-Canadine | KCNH2 |
| MOL002903 | (R)-Canadine | CHRM1 |
| MOL002903 | (R)-Canadine | SCN5A |
| MOL002903 | (R)-Canadine | F10 |
| MOL002903 | (R)-Canadine | CHRM5 |
| MOL002903 | (R)-Canadine | PTGS2 |
| MOL002903 | (R)-Canadine | ADRA2C |
| MOL002903 | (R)-Canadine | CHRM4 |
| MOL002903 | (R)-Canadine | OPRD1 |
| MOL002903 | (R)-Canadine | ADRA1B |
| MOL002903 | (R)-Canadine | SLC6A3 |
| MOL002903 | (R)-Canadine | ADRB2 |
| MOL002903 | (R)-Canadine | ADRA1D |
| MOL002903 | (R)-Canadine | SLC6A4 |
| MOL002903 | (R)-Canadine | OPRM1 |
| MOL002903 | (R)-Canadine | HSP90AA1 |
| MOL002903 | (R)-Canadine | PRKACA |
| MOL002903 | (R)-Canadine | PDE10A |
| MOL002903 | (R)-Canadine | CAMKMT |
| MOL002903 | (R)-Canadine | DRD1 |
| MOL002903 | (R)-Canadine | DRD5 |
| MOL002903 | (R)-Canadine | RXRA |
| MOL002903 | (R)-Canadine | SLC6A2 |
| MOL002903 | (R)-Canadine | ADRA1A |
| MOL002903 | (R)-Canadine | CHRM2 |
| MOL002904 | Berlambine | NOS2 |
| MOL002904 | Berlambine | PTGS1 |
| MOL002904 | Berlambine | CHRM3 |
| MOL002904 | Berlambine | KCNH2 |
| MOL002904 | Berlambine | AR |
| MOL002904 | Berlambine | SCN5A |
| MOL002904 | Berlambine | F10 |
| MOL002904 | Berlambine | PTGS2 |
| MOL002904 | Berlambine | F7 |
| MOL002904 | Berlambine | RXRA |
| MOL002904 | Berlambine | ADRA1B |
| MOL002904 | Berlambine | ADRB2 |
| MOL002904 | Berlambine | ADRA1D |
| MOL002904 | Berlambine | HSP90AA1 |
| MOL002904 | Berlambine | PRKACA |
| MOL002904 | Berlambine | PRSS1 |
| MOL002904 | Berlambine | NCOA2 |
| MOL002904 | Berlambine | CAMKMT |
| MOL002907 | Corchoroside A_qt | NR3C2 |
| MOL002907 | Corchoroside A_qt | NCOA2 |
| MOL000622 | Magnograndiolide | GRIA2 |
| MOL000098 | quercetin | PTGS1 |
| MOL000098 | quercetin | AR |
| MOL000098 | quercetin | PPARG |
| MOL000098 | quercetin | PTGS2 |
| MOL000098 | quercetin | HSP90AA1 |
| MOL000098 | quercetin | NCOA2 |
| MOL000098 | quercetin | DPP4 |
| MOL000098 | quercetin | AKR1B1 |
| MOL000098 | quercetin | PRSS1 |
| MOL000098 | quercetin | TOP2A |
| MOL000098 | quercetin | F2 |
| MOL000098 | quercetin | KCNH2 |
| MOL000098 | quercetin | SCN5A |
| MOL000098 | quercetin | F10 |
| MOL000098 | quercetin | ADRB2 |
| MOL000098 | quercetin | MMP3 |
| MOL000098 | quercetin | PRKACA |
| MOL000098 | quercetin | F7 |
| MOL000098 | quercetin | RXRA |
| MOL000098 | quercetin | ACHE |
| MOL000098 | quercetin | MAOB |
| MOL000098 | quercetin | RELA |
| MOL000098 | quercetin | EGFR |
| MOL000098 | quercetin | AKT1 |
| MOL000098 | quercetin | CCND1 |
| MOL000098 | quercetin | BCL2 |
| MOL000098 | quercetin | BCL2L1 |
| MOL000098 | quercetin | FOS |
| MOL000098 | quercetin | CDKN1A |
| MOL000098 | quercetin | EIF6 |
| MOL000098 | quercetin | BAX |
| MOL000098 | quercetin | CASP9 |
| MOL000098 | quercetin | PLAU |
| MOL000098 | quercetin | MMP2 |
| MOL000098 | quercetin | MMP9 |
| MOL000098 | quercetin | MAPK1 |
| MOL000098 | quercetin | IL10RA |
| MOL000098 | quercetin | RB1 |
| MOL000098 | quercetin | TNFAIP6 |
| MOL000098 | quercetin | JUN |
| MOL000098 | quercetin | IL6R |
| MOL000098 | quercetin | AHSA1 |
| MOL000098 | quercetin | CASP3 |
| MOL000098 | quercetin | TP53 |
| MOL000098 | quercetin | ELK1 |
| MOL000098 | quercetin | NFKBIA |
| MOL000098 | quercetin | ODC1 |
| MOL000098 | quercetin | CASP8 |
| MOL000098 | quercetin | TOP1 |
| MOL000098 | quercetin | RAF1 |
| MOL000098 | quercetin | SOD1 |
| MOL000098 | quercetin | PRKCA |
| MOL000098 | quercetin | MMP1 |
| MOL000098 | quercetin | HIF1A |
| MOL000098 | quercetin | STAT1 |
| MOL000098 | quercetin | RUNX1T1 |
| MOL000098 | quercetin | CDK1 |
| MOL000098 | quercetin | HSPA5 |
| MOL000098 | quercetin | ERBB2 |
| MOL000098 | quercetin | PPARG |
| MOL000098 | quercetin | ACACA |
| MOL000098 | quercetin | HMOX1 |
| MOL000098 | quercetin | CYP3A4 |
| MOL000098 | quercetin | CAV1 |
| MOL000098 | quercetin | MYC |
| MOL000098 | quercetin | F3 |
| MOL000098 | quercetin | GJA1 |
| MOL000098 | quercetin | CYP1A1 |
| MOL000098 | quercetin | ICAM1 |
| MOL000098 | quercetin | IL1B |
| MOL000098 | quercetin | CCL2 |
| MOL000098 | quercetin | SELE |
| MOL000098 | quercetin | VCAM1 |
| MOL000098 | quercetin | CXCL8 |
| MOL000098 | quercetin | PRKCB |
| MOL000098 | quercetin | BIRC5 |
| MOL000098 | quercetin | DUOX2 |
| MOL000098 | quercetin | NOS3 |
| MOL000098 | quercetin | HSPB1 |
| MOL000098 | quercetin | IL2RA |
| MOL000098 | quercetin | NR1I2 |
| MOL000098 | quercetin | CYP1B1 |
| MOL000098 | quercetin | CCNB1 |
| MOL000098 | quercetin | PLAT |
| MOL000098 | quercetin | THBD |
| MOL000098 | quercetin | SERPINE1 |
| MOL000098 | quercetin | IFNG |
| MOL000098 | quercetin | ALOX5 |
| MOL000098 | quercetin | IL1A |
| MOL000098 | quercetin | MPO |
| MOL000098 | quercetin | TOP2A |
| MOL000098 | quercetin | NCF1 |
| MOL000098 | quercetin | ABCG2 |
| MOL000098 | quercetin | HAS2 |
| MOL000098 | quercetin | NFE2L2 |
| MOL000098 | quercetin | NQO1 |
| MOL000098 | quercetin | PARP1 |
| MOL000098 | quercetin | AHR |
| MOL000098 | quercetin | PSMD3 |
| MOL000098 | quercetin | SLC2A4 |
| MOL000098 | quercetin | COL3A1 |
| MOL000098 | quercetin | CXCL11 |
| MOL000098 | quercetin | CXCL2 |
| MOL000098 | quercetin | DCAF5 |
| MOL000098 | quercetin | NR1I3 |
| MOL000098 | quercetin | CHEK2 |
| MOL000098 | quercetin | INSRR |
| MOL000098 | quercetin | CLDN4 |
| MOL000098 | quercetin | PPARA |
| MOL000098 | quercetin | PPARD |
| MOL000098 | quercetin | HSF1 |
| MOL000098 | quercetin | CXCL10 |
| MOL000098 | quercetin | CHUK |
| MOL000098 | quercetin | SPP1 |
| MOL000098 | quercetin | RUNX2 |
| MOL000098 | quercetin | RASSF1 |
| MOL000098 | quercetin | E2F1 |
| MOL000098 | quercetin | E2F2 |
| MOL000098 | quercetin | ACP3 |
| MOL000098 | quercetin | CTSD |
| MOL000098 | quercetin | IGFBP3 |
| MOL000098 | quercetin | IGF2 |
| MOL000098 | quercetin | CD40LG |
| MOL000098 | quercetin | IRF1 |
| MOL000098 | quercetin | ERBB3 |
| MOL000098 | quercetin | PON1 |
| MOL000098 | quercetin | DIO1 |
| MOL000098 | quercetin | PCOLCE |
| MOL000098 | quercetin | NPEPPS |
| MOL000098 | quercetin | HK2 |
| MOL000098 | quercetin | RASA1 |
| MOL000098 | quercetin | GSTM1 |
| MOL000098 | quercetin | GSTM2 |
| MOL000098 | quercetin | IL6 |
| MOL002668 | Worenine | NOS2 |
| MOL002668 | Worenine | PTGS1 |
| MOL002668 | Worenine | ESR1 |
| MOL002668 | Worenine | AR |
| MOL002668 | Worenine | PTGS2 |
| MOL002668 | Worenine | CHEK1 |
| MOL000173 | wogonin | NOS2 |
| MOL000173 | wogonin | PTGS1 |
| MOL000173 | wogonin | ESR1 |
| MOL000173 | wogonin | AR |
| MOL000173 | wogonin | SCN5A |
| MOL000173 | wogonin | PPARG |
| MOL000173 | wogonin | PTGS2 |
| MOL000173 | wogonin | RXRA |
| MOL000173 | wogonin | PDE3A |
| MOL000173 | wogonin | DPP4 |
| MOL000173 | wogonin | MAPK14 |
| MOL000173 | wogonin | GSK3B |
| MOL000173 | wogonin | HSP90AA1 |
| MOL000173 | wogonin | CDK2 |
| MOL000173 | wogonin | CHEK1 |
| MOL000173 | wogonin | PRKACA |
| MOL000173 | wogonin | PRSS1 |
| MOL000173 | wogonin | CAMKMT |
| MOL000173 | wogonin | ADRB2 |
| MOL000173 | wogonin | RELA |
| MOL000173 | wogonin | AKT1 |
| MOL000173 | wogonin | CCND1 |
| MOL000173 | wogonin | BCL2 |
| MOL000173 | wogonin | CDKN1A |
| MOL000173 | wogonin | EIF6 |
| MOL000173 | wogonin | BAX |
| MOL000173 | wogonin | CASP9 |
| MOL000173 | wogonin | KDR |
| MOL000173 | wogonin | TNFAIP6 |
| MOL000173 | wogonin | JUN |
| MOL000173 | wogonin | IL6R |
| MOL000173 | wogonin | AHSA1 |
| MOL000173 | wogonin | CASP3 |
| MOL000173 | wogonin | TP53 |
| MOL000173 | wogonin | TEP1 |
| MOL000173 | wogonin | MMP1 |
| MOL000173 | wogonin | CCL2 |
| MOL000173 | wogonin | PRKCD |
| MOL000173 | wogonin | FN1 |
| MOL000173 | wogonin | CXCL8 |
| MOL000173 | wogonin | MCL1 |
| MOL003283 | (2R,3R,4S)-4-(4-hydroxy-3-methoxy-phenyl)-7-methoxy-2,3-dimethylol-tetralin-6-ol | ESR1 |
| MOL003283 | (2R,3R,4S)-4-(4-hydroxy-3-methoxy-phenyl)-7-methoxy-2,3-dimethylol-tetralin-6-ol | AR |
| MOL003283 | (2R,3R,4S)-4-(4-hydroxy-3-methoxy-phenyl)-7-methoxy-2,3-dimethylol-tetralin-6-ol | PPARG |
| MOL003283 | (2R,3R,4S)-4-(4-hydroxy-3-methoxy-phenyl)-7-methoxy-2,3-dimethylol-tetralin-6-ol | F10 |
| MOL003283 | (2R,3R,4S)-4-(4-hydroxy-3-methoxy-phenyl)-7-methoxy-2,3-dimethylol-tetralin-6-ol | PTGS2 |
| MOL003283 | (2R,3R,4S)-4-(4-hydroxy-3-methoxy-phenyl)-7-methoxy-2,3-dimethylol-tetralin-6-ol | CA2 |
| MOL003283 | (2R,3R,4S)-4-(4-hydroxy-3-methoxy-phenyl)-7-methoxy-2,3-dimethylol-tetralin-6-ol | F7 |
| MOL003283 | (2R,3R,4S)-4-(4-hydroxy-3-methoxy-phenyl)-7-methoxy-2,3-dimethylol-tetralin-6-ol | ADRB2 |
| MOL003283 | (2R,3R,4S)-4-(4-hydroxy-3-methoxy-phenyl)-7-methoxy-2,3-dimethylol-tetralin-6-ol | TOP2A |
| MOL003283 | (2R,3R,4S)-4-(4-hydroxy-3-methoxy-phenyl)-7-methoxy-2,3-dimethylol-tetralin-6-ol | ESR2 |
| MOL003283 | (2R,3R,4S)-4-(4-hydroxy-3-methoxy-phenyl)-7-methoxy-2,3-dimethylol-tetralin-6-ol | MAPK14 |
| MOL003283 | (2R,3R,4S)-4-(4-hydroxy-3-methoxy-phenyl)-7-methoxy-2,3-dimethylol-tetralin-6-ol | GSK3B |
| MOL003283 | (2R,3R,4S)-4-(4-hydroxy-3-methoxy-phenyl)-7-methoxy-2,3-dimethylol-tetralin-6-ol | HSP90AA1 |
| MOL003283 | (2R,3R,4S)-4-(4-hydroxy-3-methoxy-phenyl)-7-methoxy-2,3-dimethylol-tetralin-6-ol | CHEK1 |
| MOL003283 | (2R,3R,4S)-4-(4-hydroxy-3-methoxy-phenyl)-7-methoxy-2,3-dimethylol-tetralin-6-ol | NCOA2 |
| MOL003283 | (2R,3R,4S)-4-(4-hydroxy-3-methoxy-phenyl)-7-methoxy-2,3-dimethylol-tetralin-6-ol | CAMKMT |
| MOL003283 | (2R,3R,4S)-4-(4-hydroxy-3-methoxy-phenyl)-7-methoxy-2,3-dimethylol-tetralin-6-ol | SCN5A |
| MOL003283 | (2R,3R,4S)-4-(4-hydroxy-3-methoxy-phenyl)-7-methoxy-2,3-dimethylol-tetralin-6-ol | CCNA2 |
| MOL003283 | (2R,3R,4S)-4-(4-hydroxy-3-methoxy-phenyl)-7-methoxy-2,3-dimethylol-tetralin-6-ol | PTGS1 |
| MOL003283 | (2R,3R,4S)-4-(4-hydroxy-3-methoxy-phenyl)-7-methoxy-2,3-dimethylol-tetralin-6-ol | IGHG1 |
| MOL003290 | (3R,4R)-3,4-bis[(3,4-dimethoxyphenyl)methyl]oxolan-2-one | CHRM3 |
| MOL003290 | (3R,4R)-3,4-bis[(3,4-dimethoxyphenyl)methyl]oxolan-2-one | KCNH2 |
| MOL003290 | (3R,4R)-3,4-bis[(3,4-dimethoxyphenyl)methyl]oxolan-2-one | ESR1 |
| MOL003290 | (3R,4R)-3,4-bis[(3,4-dimethoxyphenyl)methyl]oxolan-2-one | SCN5A |
| MOL003290 | (3R,4R)-3,4-bis[(3,4-dimethoxyphenyl)methyl]oxolan-2-one | F10 |
| MOL003290 | (3R,4R)-3,4-bis[(3,4-dimethoxyphenyl)methyl]oxolan-2-one | PTGS2 |
| MOL003290 | (3R,4R)-3,4-bis[(3,4-dimethoxyphenyl)methyl]oxolan-2-one | F7 |
| MOL003290 | (3R,4R)-3,4-bis[(3,4-dimethoxyphenyl)methyl]oxolan-2-one | PDE3A |
| MOL003290 | (3R,4R)-3,4-bis[(3,4-dimethoxyphenyl)methyl]oxolan-2-one | ADRA1B |
| MOL003290 | (3R,4R)-3,4-bis[(3,4-dimethoxyphenyl)methyl]oxolan-2-one | PTPN1 |
| MOL003290 | (3R,4R)-3,4-bis[(3,4-dimethoxyphenyl)methyl]oxolan-2-one | SLC6A3 |
| MOL003290 | (3R,4R)-3,4-bis[(3,4-dimethoxyphenyl)methyl]oxolan-2-one | ADRB2 |
| MOL003290 | (3R,4R)-3,4-bis[(3,4-dimethoxyphenyl)methyl]oxolan-2-one | ADRA1D |
| MOL003290 | (3R,4R)-3,4-bis[(3,4-dimethoxyphenyl)methyl]oxolan-2-one | HSP90AA1 |
| MOL003290 | (3R,4R)-3,4-bis[(3,4-dimethoxyphenyl)methyl]oxolan-2-one | NCOA2 |
| MOL003290 | (3R,4R)-3,4-bis[(3,4-dimethoxyphenyl)methyl]oxolan-2-one | CAMKMT |
| MOL003295 | (+)-pinoresinol monomethyl ether | PTGS1 |
| MOL003295 | (+)-pinoresinol monomethyl ether | KCNH2 |
| MOL003295 | (+)-pinoresinol monomethyl ether | SCN5A |
| MOL003295 | (+)-pinoresinol monomethyl ether | F10 |
| MOL003295 | (+)-pinoresinol monomethyl ether | PTGS2 |
| MOL003295 | (+)-pinoresinol monomethyl ether | RXRA |
| MOL003295 | (+)-pinoresinol monomethyl ether | PDE3A |
| MOL003295 | (+)-pinoresinol monomethyl ether | ADRA1B |
| MOL003295 | (+)-pinoresinol monomethyl ether | ADRB2 |
| MOL003295 | (+)-pinoresinol monomethyl ether | HSP90AA1 |
| MOL003295 | (+)-pinoresinol monomethyl ether | NPPB |
| MOL003295 | (+)-pinoresinol monomethyl ether | NCOA2 |
| MOL003295 | (+)-pinoresinol monomethyl ether | NCOA1 |
| MOL003295 | (+)-pinoresinol monomethyl ether | CAMKMT |
| MOL003305 | PHILLYRIN | TOP2A |
| MOL003306 | ACon1_001697 | PTGS1 |
| MOL003306 | ACon1_001697 | KCNH2 |
| MOL003306 | ACon1_001697 | SCN5A |
| MOL003306 | ACon1_001697 | F10 |
| MOL003306 | ACon1_001697 | PTGS2 |
| MOL003306 | ACon1_001697 | ADRA1B |
| MOL003306 | ACon1_001697 | ADRB2 |
| MOL003306 | ACon1_001697 | HSP90AA1 |
| MOL003306 | ACon1_001697 | PRKACA |
| MOL003306 | ACon1_001697 | NCOA2 |
| MOL003306 | ACon1_001697 | NCOA1 |
| MOL003306 | ACon1_001697 | CAMKMT |
| MOL003308 | (+)-pinoresinol monomethyl ether-4-D-beta-glucoside_qt | KCNH2 |
| MOL003308 | (+)-pinoresinol monomethyl ether-4-D-beta-glucoside_qt | SCN5A |
| MOL003308 | (+)-pinoresinol monomethyl ether-4-D-beta-glucoside_qt | F10 |
| MOL003308 | (+)-pinoresinol monomethyl ether-4-D-beta-glucoside_qt | PTGS2 |
| MOL003308 | (+)-pinoresinol monomethyl ether-4-D-beta-glucoside_qt | ADRB2 |
| MOL003308 | (+)-pinoresinol monomethyl ether-4-D-beta-glucoside_qt | HSP90AA1 |
| MOL003308 | (+)-pinoresinol monomethyl ether-4-D-beta-glucoside_qt | PRKACA |
| MOL003308 | (+)-pinoresinol monomethyl ether-4-D-beta-glucoside_qt | NCOA2 |
| MOL003308 | (+)-pinoresinol monomethyl ether-4-D-beta-glucoside_qt | NCOA1 |
| MOL003308 | (+)-pinoresinol monomethyl ether-4-D-beta-glucoside_qt | CAMKMT |
| MOL003315 | 3beta-Acetyl-20,25-epoxydammarane-24alpha-ol | NR3C1 |
| MOL000211 | Mairin | PGR |
| MOL003322 | FORSYTHINOL | KCNH2 |
| MOL003322 | FORSYTHINOL | SCN5A |
| MOL003322 | FORSYTHINOL | F10 |
| MOL003322 | FORSYTHINOL | PTGS2 |
| MOL003322 | FORSYTHINOL | ADRA1B |
| MOL003322 | FORSYTHINOL | ADRB2 |
| MOL003322 | FORSYTHINOL | HSP90AA1 |
| MOL003322 | FORSYTHINOL | NCOA2 |
| MOL003322 | FORSYTHINOL | NCOA1 |
| MOL003322 | FORSYTHINOL | CAMKMT |
| MOL003330 | (-)-Phillygenin | CHRM3 |
| MOL003330 | (-)-Phillygenin | KCNH2 |
| MOL003330 | (-)-Phillygenin | CHRM1 |
| MOL003330 | (-)-Phillygenin | SCN5A |
| MOL003330 | (-)-Phillygenin | F10 |
| MOL003330 | (-)-Phillygenin | CHRM5 |
| MOL003330 | (-)-Phillygenin | PTGS2 |
| MOL003330 | (-)-Phillygenin | ADRA1B |
| MOL003330 | (-)-Phillygenin | ADRB2 |
| MOL003330 | (-)-Phillygenin | HSP90AA1 |
| MOL003330 | (-)-Phillygenin | IGHG1 |
| MOL003330 | (-)-Phillygenin | NCOA2 |
| MOL003330 | (-)-Phillygenin | CAMKMT |
| MOL003347 | hyperforin | CYP3A4 |
| MOL003347 | hyperforin | ICAM1 |
| MOL003347 | hyperforin | CXCL8 |
| MOL003347 | hyperforin | NR1I2 |
| MOL003370 | Onjixanthone I | NOS2 |
| MOL003370 | Onjixanthone I | PTGS1 |
| MOL003370 | Onjixanthone I | SCN5A |
| MOL003370 | Onjixanthone I | PTGS2 |
| MOL003370 | Onjixanthone I | RXRA |
| MOL003370 | Onjixanthone I | ESR2 |
| MOL003370 | Onjixanthone I | DPP4 |
| MOL003370 | Onjixanthone I | HSP90AA1 |
| MOL003370 | Onjixanthone I | CHEK1 |
| MOL003370 | Onjixanthone I | CAMKMT |
| MOL000358 | beta-sitosterol | PGR |
| MOL000358 | beta-sitosterol | NCOA2 |
| MOL000358 | beta-sitosterol | PTGS1 |
| MOL000358 | beta-sitosterol | PTGS2 |
| MOL000358 | beta-sitosterol | HSP90AA1 |
| MOL000358 | beta-sitosterol | KCNH2 |
| MOL000358 | beta-sitosterol | PRKACA |
| MOL000358 | beta-sitosterol | DRD1 |
| MOL000358 | beta-sitosterol | CHRM3 |
| MOL000358 | beta-sitosterol | CHRM1 |
| MOL000358 | beta-sitosterol | SCN5A |
| MOL000358 | beta-sitosterol | CHRM4 |
| MOL000358 | beta-sitosterol | PDE3A |
| MOL000358 | beta-sitosterol | ADRA1A |
| MOL000358 | beta-sitosterol | CHRM2 |
| MOL000358 | beta-sitosterol | ADRA1B |
| MOL000358 | beta-sitosterol | ADRB2 |
| MOL000358 | beta-sitosterol | CHRNA2 |
| MOL000358 | beta-sitosterol | SLC6A4 |
| MOL000358 | beta-sitosterol | OPRM1 |
| MOL000358 | beta-sitosterol | CHRNA7 |
| MOL000358 | beta-sitosterol | BCL2 |
| MOL000358 | beta-sitosterol | BAX |
| MOL000358 | beta-sitosterol | CASP9 |
| MOL000358 | beta-sitosterol | JUN |
| MOL000358 | beta-sitosterol | CASP3 |
| MOL000358 | beta-sitosterol | CASP8 |
| MOL000358 | beta-sitosterol | PRKCA |
| MOL000358 | beta-sitosterol | PON1 |
| MOL000358 | beta-sitosterol | MAP2 |
| MOL000422 | kaempferol | NOS2 |
| MOL000422 | kaempferol | PTGS1 |
| MOL000422 | kaempferol | AR |
| MOL000422 | kaempferol | PPARG |
| MOL000422 | kaempferol | PTGS2 |
| MOL000422 | kaempferol | HSP90AA1 |
| MOL000422 | kaempferol | PRKACA |
| MOL000422 | kaempferol | NCOA2 |
| MOL000422 | kaempferol | DPP4 |
| MOL000422 | kaempferol | PRSS1 |
| MOL000422 | kaempferol | PGR |
| MOL000422 | kaempferol | F2 |
| MOL000422 | kaempferol | CHRM1 |
| MOL000422 | kaempferol | ACHE |
| MOL000422 | kaempferol | SLC6A2 |
| MOL000422 | kaempferol | CHRM2 |
| MOL000422 | kaempferol | ADRA1B |
| MOL000422 | kaempferol | TOP2A |
| MOL000422 | kaempferol | F7 |
| MOL000422 | kaempferol | CAMKMT |
| MOL000422 | kaempferol | RELA |
| MOL000422 | kaempferol | IKBKB |
| MOL000422 | kaempferol | AKT1 |
| MOL000422 | kaempferol | BCL2 |
| MOL000422 | kaempferol | BAX |
| MOL000422 | kaempferol | TNFAIP6 |
| MOL000422 | kaempferol | JUN |
| MOL000422 | kaempferol | AHSA1 |
| MOL000422 | kaempferol | CASP3 |
| MOL000422 | kaempferol | MAPK8 |
| MOL000422 | kaempferol | MMP1 |
| MOL000422 | kaempferol | STAT1 |
| MOL000422 | kaempferol | CDK1 |
| MOL000422 | kaempferol | PPARG |
| MOL000422 | kaempferol | HMOX1 |
| MOL000422 | kaempferol | CYP3A4 |
| MOL000422 | kaempferol | CYP1A1 |
| MOL000422 | kaempferol | ICAM1 |
| MOL000422 | kaempferol | SELE |
| MOL000422 | kaempferol | VCAM1 |
| MOL000422 | kaempferol | NR1I2 |
| MOL000422 | kaempferol | CYP1B1 |
| MOL000422 | kaempferol | ALOX5 |
| MOL000422 | kaempferol | HAS2 |
| MOL000422 | kaempferol | AHR |
| MOL000422 | kaempferol | PSMD3 |
| MOL000422 | kaempferol | SLC2A4 |
| MOL000422 | kaempferol | NR1I3 |
| MOL000422 | kaempferol | INSRR |
| MOL000422 | kaempferol | DIO1 |
| MOL000422 | kaempferol | PPP3CA |
| MOL000422 | kaempferol | GSTM1 |
| MOL000422 | kaempferol | GSTM2 |
| MOL000422 | kaempferol | AKR1C3 |
| MOL000422 | kaempferol | SLPI |
| MOL000522 | arctiin | KCNH2 |
| MOL000522 | arctiin | SCN5A |
| MOL000522 | arctiin | F10 |
| MOL000522 | arctiin | PTGS2 |
| MOL000522 | arctiin | KDR |
| MOL000522 | arctiin | PTPN1 |
| MOL000522 | arctiin | ADRB2 |
| MOL000522 | arctiin | HSP90AA1 |
| MOL000522 | arctiin | NCOA1 |
| MOL000522 | arctiin | MUC1 |
| MOL000006 | luteolin | PTGS1 |
| MOL000006 | luteolin | AR |
| MOL000006 | luteolin | PTGS2 |
| MOL000006 | luteolin | HSP90AA1 |
| MOL000006 | luteolin | PRSS1 |
| MOL000006 | luteolin | NCOA2 |
| MOL000006 | luteolin | PRKACA |
| MOL000006 | luteolin | DPP4 |
| MOL000006 | luteolin | RELA |
| MOL000006 | luteolin | EGFR |
| MOL000006 | luteolin | AKT1 |
| MOL000006 | luteolin | CCND1 |
| MOL000006 | luteolin | BCL2L1 |
| MOL000006 | luteolin | CDKN1A |
| MOL000006 | luteolin | CASP9 |
| MOL000006 | luteolin | MMP2 |
| MOL000006 | luteolin | MMP9 |
| MOL000006 | luteolin | MAPK1 |
| MOL000006 | luteolin | IL10RA |
| MOL000006 | luteolin | RB1 |
| MOL000006 | luteolin | CDK4 |
| MOL000006 | luteolin | TNFAIP6 |
| MOL000006 | luteolin | JUN |
| MOL000006 | luteolin | IL6R |
| MOL000006 | luteolin | CASP3 |
| MOL000006 | luteolin | TP53 |
| MOL000006 | luteolin | NFKBIA |
| MOL000006 | luteolin | TOP1 |
| MOL000006 | luteolin | MDM2 |
| MOL000006 | luteolin | APP |
| MOL000006 | luteolin | MMP1 |
| MOL000006 | luteolin | PCNA |
| MOL000006 | luteolin | ERBB2 |
| MOL000006 | luteolin | PPARG |
| MOL000006 | luteolin | HMOX1 |
| MOL000006 | luteolin | CASP7 |
| MOL000006 | luteolin | ICAM1 |
| MOL000006 | luteolin | MCL1 |
| MOL000006 | luteolin | BIRC5 |
| MOL000006 | luteolin | IL2RA |
| MOL000006 | luteolin | CCNB1 |
| MOL000006 | luteolin | TYR |
| MOL000006 | luteolin | IFNG |
| MOL000006 | luteolin | IL4 |
| MOL000006 | luteolin | TOP2A |
| MOL000006 | luteolin | XIAP |
| MOL000006 | luteolin | SLC2A4 |
| MOL000006 | luteolin | INSRR |
| MOL000006 | luteolin | CD40LG |
| MOL000006 | luteolin | PTGES |
| MOL000006 | luteolin | NUF2 |
| MOL000006 | luteolin | ADCY2 |
| MOL000006 | luteolin | MET |
| MOL000791 | bicuculline | PTGS1 |
| MOL000791 | bicuculline | F2 |
| MOL000791 | bicuculline | KCNH2 |
| MOL000791 | bicuculline | AR |
| MOL000791 | bicuculline | SCN5A |
| MOL000791 | bicuculline | F10 |
| MOL000791 | bicuculline | PTGS2 |
| MOL000791 | bicuculline | KDR |
| MOL000791 | bicuculline | ACHE |
| MOL000791 | bicuculline | TOP2A |
| MOL000791 | bicuculline | HSP90AA1 |
| MOL000791 | bicuculline | PTPN1 |
| MOL000791 | bicuculline | PRKACA |
| MOL000791 | bicuculline | FOS |
| MOL000791 | bicuculline | GJA1 |
| MOL000791 | bicuculline | GABBR1 |
| MOL000791 | bicuculline | BMPR2 |
| MOL000791 | bicuculline | GRM5 |
| MOL000791 | bicuculline | GNRH1 |
| MOL000791 | bicuculline | GNRHR |
| MOL000791 | bicuculline | CRH |
| MOL000791 | bicuculline | GRIN2D |
| MOL000791 | bicuculline | SLC6A2 |
| MOL000791 | bicuculline | GJB1 |
| MOL000791 | bicuculline | GRM1 |
| MOL000791 | bicuculline | VCP |
| MOL000098 | quercetin | PTGS1 |
| MOL000098 | quercetin | AR |
| MOL000098 | quercetin | PPARG |
| MOL000098 | quercetin | PTGS2 |
| MOL000098 | quercetin | HSP90AA1 |
| MOL000098 | quercetin | NCOA2 |
| MOL000098 | quercetin | DPP4 |
| MOL000098 | quercetin | AKR1B1 |
| MOL000098 | quercetin | PRSS1 |
| MOL000098 | quercetin | TOP2A |
| MOL000098 | quercetin | F2 |
| MOL000098 | quercetin | KCNH2 |
| MOL000098 | quercetin | SCN5A |
| MOL000098 | quercetin | F10 |
| MOL000098 | quercetin | ADRB2 |
| MOL000098 | quercetin | MMP3 |
| MOL000098 | quercetin | PRKACA |
| MOL000098 | quercetin | F7 |
| MOL000098 | quercetin | RXRA |
| MOL000098 | quercetin | ACHE |
| MOL000098 | quercetin | MAOB |
| MOL000098 | quercetin | RELA |
| MOL000098 | quercetin | EGFR |
| MOL000098 | quercetin | AKT1 |
| MOL000098 | quercetin | NPPB |
| MOL000098 | quercetin | CCND1 |
| MOL000098 | quercetin | BCL2 |
| MOL000098 | quercetin | BCL2L1 |
| MOL000098 | quercetin | FOS |
| MOL000098 | quercetin | CDKN1A |
| MOL000098 | quercetin | EIF6 |
| MOL000098 | quercetin | BAX |
| MOL000098 | quercetin | CASP9 |
| MOL000098 | quercetin | PLAU |
| MOL000098 | quercetin | MMP2 |
| MOL000098 | quercetin | MMP9 |
| MOL000098 | quercetin | MAPK1 |
| MOL000098 | quercetin | IL10RA |
| MOL000098 | quercetin | RB1 |
| MOL000098 | quercetin | TNFAIP6 |
| MOL000098 | quercetin | JUN |
| MOL000098 | quercetin | IL6R |
| MOL000098 | quercetin | AHSA1 |
| MOL000098 | quercetin | CASP3 |
| MOL000098 | quercetin | TP53 |
| MOL000098 | quercetin | ELK1 |
| MOL000098 | quercetin | NFKBIA |
| MOL000098 | quercetin | ODC1 |
| MOL000098 | quercetin | CASP8 |
| MOL000098 | quercetin | TOP1 |
| MOL000098 | quercetin | RAF1 |
| MOL000098 | quercetin | SOD1 |
| MOL000098 | quercetin | PRKCA |
| MOL000098 | quercetin | MMP1 |
| MOL000098 | quercetin | HIF1A |
| MOL000098 | quercetin | STAT1 |
| MOL000098 | quercetin | RUNX1T1 |
| MOL000098 | quercetin | CDK1 |
| MOL000098 | quercetin | HSPA5 |
| MOL000098 | quercetin | ERBB2 |
| MOL000098 | quercetin | PPARG |
| MOL000098 | quercetin | ACACA |
| MOL000098 | quercetin | HMOX1 |
| MOL000098 | quercetin | CYP3A4 |
| MOL000098 | quercetin | CAV1 |
| MOL000098 | quercetin | MYC |
| MOL000098 | quercetin | F3 |
| MOL000098 | quercetin | GJA1 |
| MOL000098 | quercetin | CYP1A1 |
| MOL000098 | quercetin | ICAM1 |
| MOL000098 | quercetin | IL1B |
| MOL000098 | quercetin | CCL2 |
| MOL000098 | quercetin | SELE |
| MOL000098 | quercetin | VCAM1 |
| MOL000098 | quercetin | CXCL8 |
| MOL000098 | quercetin | PRKCB |
| MOL000098 | quercetin | BIRC5 |
| MOL000098 | quercetin | DUOX2 |
| MOL000098 | quercetin | NOS3 |
| MOL000098 | quercetin | HSPB1 |
| MOL000098 | quercetin | IL2RA |
| MOL000098 | quercetin | NR1I2 |
| MOL000098 | quercetin | CYP1B1 |
| MOL000098 | quercetin | CCNB1 |
| MOL000098 | quercetin | PLAT |
| MOL000098 | quercetin | THBD |
| MOL000098 | quercetin | SERPINE1 |
| MOL000098 | quercetin | IFNG |
| MOL000098 | quercetin | ALOX5 |
| MOL000098 | quercetin | IL1A |
| MOL000098 | quercetin | MPO |
| MOL000098 | quercetin | TOP2A |
| MOL000098 | quercetin | NCF1 |
| MOL000098 | quercetin | ABCG2 |
| MOL000098 | quercetin | HAS2 |
| MOL000098 | quercetin | NFE2L2 |
| MOL000098 | quercetin | NQO1 |
| MOL000098 | quercetin | PARP1 |
| MOL000098 | quercetin | AHR |
| MOL000098 | quercetin | PSMD3 |
| MOL000098 | quercetin | SLC2A4 |
| MOL000098 | quercetin | COL3A1 |
| MOL000098 | quercetin | CXCL11 |
| MOL000098 | quercetin | CXCL2 |
| MOL000098 | quercetin | DCAF5 |
| MOL000098 | quercetin | NR1I3 |
| MOL000098 | quercetin | CHEK2 |
| MOL000098 | quercetin | INSRR |
| MOL000098 | quercetin | CLDN4 |
| MOL000098 | quercetin | PPARA |
| MOL000098 | quercetin | PPARD |
| MOL000098 | quercetin | HSF1 |
| MOL000098 | quercetin | CXCL10 |
| MOL000098 | quercetin | CHUK |
| MOL000098 | quercetin | SPP1 |
| MOL000098 | quercetin | RUNX2 |
| MOL000098 | quercetin | RASSF1 |
| MOL000098 | quercetin | E2F1 |
| MOL000098 | quercetin | E2F2 |
| MOL000098 | quercetin | ACP3 |
| MOL000098 | quercetin | CTSD |
| MOL000098 | quercetin | IGFBP3 |
| MOL000098 | quercetin | IGF2 |
| MOL000098 | quercetin | CD40LG |
| MOL000098 | quercetin | IRF1 |
| MOL000098 | quercetin | ERBB3 |
| MOL000098 | quercetin | PON1 |
| MOL000098 | quercetin | DIO1 |
| MOL000098 | quercetin | PCOLCE |
| MOL000098 | quercetin | NPEPPS |
| MOL000098 | quercetin | HK2 |
| MOL000098 | quercetin | RASA1 |
| MOL000098 | quercetin | GSTM1 |
| MOL000098 | quercetin | GSTM2 |
| MOL000098 | quercetin | IL6 |
| MOL001729 | Crysophanol | ELANE |
| MOL001729 | Crysophanol | ESR2 |
| MOL001729 | Crysophanol | CSNK2A1 |
| MOL001729 | Crysophanol | PTP4A3 |
| MOL001729 | Crysophanol | PIM1 |
| MOL001729 | Crysophanol | ESR1 |
| MOL001729 | Crysophanol | FTO |
| MOL001729 | Crysophanol | MCL1 |
| MOL001729 | Crysophanol | BCL2 |
| MOL001729 | Crysophanol | CYP19A1 |
| MOL001729 | Crysophanol | FNTA |
| MOL001729 | Crysophanol | EGFR |
| MOL001729 | Crysophanol | LIMK1 |
| MOL001729 | Crysophanol | LCK |
| MOL001729 | Crysophanol | LDHB |
| MOL001729 | Crysophanol | ERN1 |
| MOL001729 | Crysophanol | CISD1 |
| MOL001729 | Crysophanol | DUSP3 |
| MOL001729 | Crysophanol | CA7 |
| MOL001729 | Crysophanol | CA14 |
| MOL001729 | Crysophanol | PLEC |
| MOL001729 | Crysophanol | CSNK1A1 |
| MOL001729 | Crysophanol | CSNK1D |
| MOL001729 | Crysophanol | LDHA |
| MOL001729 | Crysophanol | MMP16 |
| MOL001729 | Crysophanol | MMP13 |
| MOL001729 | Crysophanol | MMP3 |
| MOL001729 | Crysophanol | MMP9 |
| MOL001729 | Crysophanol | MMP1 |
| MOL001729 | Crysophanol | MMP2 |
| MOL001729 | Crysophanol | MMP14 |
| MOL001729 | Crysophanol | MMP12 |
| MOL001729 | Crysophanol | MMP8 |
| MOL001729 | Crysophanol | HTR2B |
| MOL001729 | Crysophanol | HMGCR |
| MOL001729 | Crysophanol | PIK3CG |
| MOL001729 | Crysophanol | PLAA |
| MOL001729 | Crysophanol | HDAC6 |
| MOL001729 | Crysophanol | HDAC8 |
| MOL001729 | Crysophanol | HDAC1 |
| MOL001729 | Crysophanol | HDAC5 |
| MOL001729 | Crysophanol | HDAC7 |
| MOL001729 | Crysophanol | CTSV |
| MOL001729 | Crysophanol | HDAC4 |
| MOL001729 | Crysophanol | GUSB |
| MOL001729 | Crysophanol | ADAM17 |
| MOL001729 | Crysophanol | MMP7 |
| MOL001729 | Crysophanol | MAP2K1 |
| MOL001729 | Crysophanol | NOX4 |
| MOL001729 | Crysophanol | PLA2G7 |
| MOL001729 | Crysophanol | ABL1 |
| MOL001729 | Crysophanol | SRC |
| MOL001729 | Crysophanol | KDR |
| MOL001729 | Crysophanol | MTOR |
| MOL001729 | Crysophanol | PIK3CD |
| MOL001729 | Crysophanol | PRKDC |
| MOL001729 | Crysophanol | PIK3CB |
| MOL001729 | Crysophanol | HCK |
| MOL001729 | Crysophanol | PI4KB |
| MOL001729 | Crysophanol | PIK3CA |
| MOL001729 | Crysophanol | CHEK1 |
| MOL001729 | Crysophanol | NOS2 |
| MOL001729 | Crysophanol | MAOB |
| MOL001729 | Crysophanol | GRM5 |
| MOL001729 | Crysophanol | ALPG |
| MOL001729 | Crysophanol | ALPL |
| MOL001729 | Crysophanol | HSP90AA1 |
| MOL001729 | Crysophanol | HSP90AB1 |
| MOL001729 | Crysophanol | DAO |
| MOL001729 | Crysophanol | EIF2AK2 |
| MOL001729 | Crysophanol | ACE |
| MOL001729 | Crysophanol | PTGS1 |
| MOL001729 | Crysophanol | PTGS2 |
| MOL001729 | Crysophanol | NCOA2 |
| MOL001729 | Crysophanol | CAMKMT |
| MOL001729 | Crysophanol | SCN5A |
| MOL001729 | Crysophanol | PDE3A |
| MOL001729 | Crysophanol | PRKACA |
| MOL001729 | Crysophanol | IGHG1 |
| MOL001729 | Crysophanol | PKIA |
| MOL001454 | berberine | ACHE |
| MOL001454 | berberine | HTR2B |
| MOL001454 | berberine | BCHE |
| MOL001454 | berberine | ADRA2C |
| MOL001454 | berberine | ADRA2B |
| MOL001454 | berberine | CHRM1 |
| MOL001454 | berberine | SIGMAR1 |
| MOL001454 | berberine | CYP2D6 |
| MOL001454 | berberine | SAE1 UBA2 |
| MOL001454 | berberine | RAC1 |
| MOL001454 | berberine | CDC42 |
| MOL001454 | berberine | RPS6KB1 |
| MOL001454 | berberine | AURKA |
| MOL001454 | berberine | AURKB |
| MOL001454 | berberine | CYP11B2 |
| MOL001454 | berberine | PRF1 |
| MOL001454 | berberine | GRIA1 |
| MOL001454 | berberine | TBXAS1 |
| MOL001454 | berberine | HPGD |
| MOL001454 | berberine | SLC1A3 |
| MOL001454 | berberine | GABRB3 |
| MOL001454 | berberine | PIM1 |
| MOL001454 | berberine | PIM2 |
| MOL001454 | berberine | MET |
| MOL001454 | berberine | HTR3A |
| MOL001454 | berberine | GRK5 |
| MOL001454 | berberine | CYP11B1 |
| MOL001454 | berberine | IMPDH2 |
| MOL001454 | berberine | PIK3CA |
| MOL001454 | berberine | F3 |
| MOL001454 | berberine | CYP19A1 |
| MOL001454 | berberine | BCAT2 |
| MOL001454 | berberine | TRPM8 |
| MOL001454 | berberine | ICAM1 |
| MOL001454 | berberine | SELE |
| MOL001454 | berberine | MAOB |
| MOL001454 | berberine | PIK3CD |
| MOL001454 | berberine | PIK3CB |
| MOL001454 | berberine | PIK3CG |
| MOL001454 | berberine | MAPKAPK2 |
| MOL001454 | berberine | PARP10 |
| MOL001454 | berberine | CHEK2 |
| MOL001454 | berberine | CCNC |
| MOL001454 | berberine | CDK8 |
| MOL001454 | berberine | MAPK10 |
| MOL001454 | berberine | AGPAT2 |
| MOL001454 | berberine | TGM2 |
| MOL001454 | berberine | SCD |
| MOL001454 | berberine | XBP1 |
| MOL001454 | berberine | TYMS |
| MOL001454 | berberine | DHFR |
| MOL001454 | berberine | GABRA2 |
| MOL001454 | berberine | ROCK1 |
| MOL001454 | berberine | PRKACA |
| MOL001454 | berberine | CHRM4 |
| MOL001454 | berberine | JAK2 |
| MOL001454 | berberine | LCK |
| MOL001454 | berberine | CDK9 |
| MOL001454 | berberine | PTPN1 |
| MOL001454 | berberine | ALOX5AP |
| MOL001454 | berberine | CHEK1 |
| MOL001454 | berberine | KIT |
| MOL001454 | berberine | SRC |
| MOL001454 | berberine | MKNK1 |
| MOL001454 | berberine | IKBKB |
| MOL001454 | berberine | MAPK14 |
| MOL001454 | berberine | LRRK2 |
| MOL001454 | berberine | ROCK2 |
| MOL001454 | berberine | ATR |
| MOL001454 | berberine | AOC3 |
| MOL001454 | berberine | GRK3 |
| MOL001454 | berberine | PLK1 |
| MOL001454 | berberine | GRK2 |
| MOL001454 | berberine | NPY5R |
| MOL001454 | berberine | MAP4K4 |
| MOL001454 | berberine | NTRK1 |
| MOL001454 | berberine | ABL1 |
| MOL001454 | berberine | CDK1 |
| MOL001454 | berberine | NR3C2 |
| MOL001454 | berberine | PGR |
| MOL001454 | berberine | DRD4 |
| MOL001454 | berberine | PTGS2 |
| MOL001454 | berberine | DRD3 |
| MOL001454 | berberine | CDK2 |
| MOL001454 | berberine | CDK4 |
| MOL001454 | berberine | SIRT2 |
| MOL001454 | berberine | ADORA2A |
| MOL001454 | berberine | ADORA3 |
| MOL001454 | berberine | GRM5 |
| MOL001454 | berberine | QPCT |
| MOL001454 | berberine | CSF1R |
| MOL001454 | berberine | CNR2 |
| MOL001454 | berberine | CBFB |
| MOL001454 | berberine | HSD17B1 |
| MOL001454 | berberine | PNMT |
| MOL001454 | berberine | PARP2 |
| MOL001454 | berberine | PTGES |
| MOL001454 | berberine | RPS27 |
| MOL001454 | berberine | NOS2 |
| MOL001454 | berberine | PTGS1 |
| MOL001454 | berberine | KCNH2 |
| MOL001454 | berberine | ESR1 |
| MOL001454 | berberine | AR |
| MOL001454 | berberine | SCN5A |
| MOL001454 | berberine | F10 |
| MOL001454 | berberine | PTGS2 |
| MOL001454 | berberine | RXRA |
| MOL001454 | berberine | ADRB2 |
| MOL001454 | berberine | HSP90AA1 |
| MOL001454 | berberine | PRKACA |
| MOL001454 | berberine | PRSS1 |
| MOL001454 | berberine | NCOA2 |
| MOL001454 | berberine | PDE10A |
| MOL001454 | berberine | CAMKMT |
| MOL001454 | berberine | IL6 |
| MOL001454 | berberine | TNF |
| MOL001454 | berberine | CASP3 |
| MOL001454 | berberine | COX-2 |
| MOL000785 | palmatine | ACHE |
| MOL000785 | palmatine | HTR2B |
| MOL000785 | palmatine | BCHE |
| MOL000785 | palmatine | ADRA2C |
| MOL000785 | palmatine | ADRA2B |
| MOL000785 | palmatine | CHRM1 |
| MOL000785 | palmatine | SIGMAR1 |
| MOL000785 | palmatine | CYP2D6 |
| MOL000785 | palmatine | SAE1 |
| MOL000785 | palmatine | RAC1 |
| MOL000785 | palmatine | CDC42 |
| MOL000785 | palmatine | MAP2K1 |
| MOL000785 | palmatine | AURKB |
| MOL000785 | palmatine | AURKA |
| MOL000785 | palmatine | CDK2 |
| MOL000785 | palmatine | PLK1 |
| MOL000785 | palmatine | TTK |
| MOL000785 | palmatine | PLK3 |
| MOL000785 | palmatine | PLK2 |
| MOL000785 | palmatine | PGK1 |
| MOL000785 | palmatine | TRPC6 |
| MOL000785 | palmatine | MAPKAPK2 |
| MOL000785 | palmatine | CCNC |
| MOL000785 | palmatine | CDK8 |
| MOL000785 | palmatine | HPGD |
| MOL000785 | palmatine | DYRK1A |
| MOL000785 | palmatine | GRM5 |
| MOL000785 | palmatine | HTR3A |
| MOL000785 | palmatine | CYP1A1 |
| MOL000785 | palmatine | NQO1 |
| MOL000785 | palmatine | NQO2 |
| MOL000785 | palmatine | CYP1B1 |
| MOL000785 | palmatine | DRD4 |
| MOL000785 | palmatine | PRF1 |
| MOL000785 | palmatine | EPHX2 |
| MOL000785 | palmatine | NTRK1 |
| MOL000785 | palmatine | MTOR |
| MOL000785 | palmatine | MARK1 |
| MOL000785 | palmatine | PIK3CD |
| MOL000785 | palmatine | PIK3CB |
| MOL000785 | palmatine | PIK3CG |
| MOL000785 | palmatine | KDM5B |
| MOL000785 | palmatine | ERBB2 |
| MOL000785 | palmatine | GRM1 |
| MOL000785 | palmatine | ABCG2 |
| MOL000785 | palmatine | DHFR |
| MOL000785 | palmatine | CLK4 |
| MOL000785 | palmatine | NAAA |
| MOL000785 | palmatine | PPIA |
| MOL000785 | palmatine | LCK |
| MOL000785 | palmatine | MET |
| MOL000785 | palmatine | LYN |
| MOL000785 | palmatine | EPHB4 |
| MOL000785 | palmatine | TBXAS1 |
| MOL000785 | palmatine | TEK |
| MOL000785 | palmatine | CDC25B |
| MOL000785 | palmatine | SCD |
| MOL000785 | palmatine | KIT |
| MOL000785 | palmatine | PIM1 |
| MOL000785 | palmatine | CYP11B2 |
| MOL000785 | palmatine | SLC1A3 |
| MOL000785 | palmatine | CLK1 |
| MOL000785 | palmatine | DYRK2 |
| MOL000785 | palmatine | MDM2 |
| MOL000785 | palmatine | CCND1 |
| MOL000785 | palmatine | GCK |
| MOL000785 | palmatine | ALDH2 |
| MOL000785 | palmatine | MAPK8 |
| MOL000785 | palmatine | MAPK10 |
| MOL000785 | palmatine | MCHR1 |
| MOL000785 | palmatine | HCRTR2 |
| MOL000785 | palmatine | TRPV1 |
| MOL000785 | palmatine | CSF1R |
| MOL000785 | palmatine | CFD |
| MOL000785 | palmatine | ROCK2 |
| MOL000785 | palmatine | RAF1 |
| MOL000785 | palmatine | IKBKB |
| MOL000785 | palmatine | HSD17B2 |
| MOL000785 | palmatine | F3 |
| MOL000785 | palmatine | FPR2 |
| MOL000785 | palmatine | LRRK2 |
| MOL000785 | palmatine | RPS6KA2 |
| MOL000785 | palmatine | NAMPT |
| MOL000785 | palmatine | MST1R |
| MOL000785 | palmatine | CSNK1D |
| MOL000785 | palmatine | MMP3 |
| MOL000785 | palmatine | CDK2 |
| MOL000785 | palmatine | MMP9 |
| MOL000785 | palmatine | CDK4 |
| MOL000785 | palmatine | MMP1 |
| MOL000785 | palmatine | SIRT2 |
| MOL000785 | palmatine | FLT4 |
| MOL000785 | palmatine | RET |
| MOL000785 | palmatine | ITK |
| MOL000785 | palmatine | HSD17B1 |
| MOL000785 | palmatine | STAT3 |
| MOL000785 | palmatine | HSD11B1 |
| MOL000785 | palmatine | PTGER2 |
| MOL000785 | palmatine | PDGFRB |
| MOL000785 | palmatine | PFKFB3 |
| MOL000785 | palmatine | NOS2 |
| MOL000785 | palmatine | PTGS1 |
| MOL000785 | palmatine | KCNH2 |
| MOL000785 | palmatine | ESR1 |
| MOL000785 | palmatine | AR |
| MOL000785 | palmatine | SCN5A |
| MOL000785 | palmatine | PTGS2 |
| MOL000785 | palmatine | RXRA |
| MOL000785 | palmatine | ADRB2 |
| MOL000785 | palmatine | ESR2 |
| MOL000785 | palmatine | HSP90AA1 |
| MOL000785 | palmatine | PRSS1 |
| MOL000785 | palmatine | NCOA2 |
| MOL000785 | palmatine | CAMKMT |
| MOL000785 | palmatine | PRKACA |
| MOL000785 | palmatine | CDK2 |
| MOL000785 | palmatine | F7 |
| MOL000785 | palmatine | IL6 |
| MOL000785 | palmatine | TNF |
| MOL002897 | epiberberine | SAE1 |
| MOL002897 | epiberberine | UBA2 |
| MOL002897 | epiberberine | ACHE |
| MOL002897 | epiberberine | BCHE |
| MOL002897 | epiberberine | SIGMAR1 |
| MOL002897 | epiberberine | HTR2B |
| MOL002897 | epiberberine | ADRA2C |
| MOL002897 | epiberberine | ADRA2B |
| MOL002897 | epiberberine | CHRM1 |
| MOL002897 | epiberberine | CYP2D6 |
| MOL002897 | epiberberine | RAC1 |
| MOL002897 | epiberberine | RPS6KB1 |
| MOL002897 | epiberberine | AURKA |
| MOL002897 | epiberberine | CDC42 |
| MOL002897 | epiberberine | HPGD |
| MOL002897 | epiberberine | TBXAS1 |
| MOL002897 | epiberberine | MAOB |
| MOL002897 | epiberberine | PIK3CG |
| MOL002897 | epiberberine | GRK5 |
| MOL002897 | epiberberine | CYP19A1 |
| MOL002897 | epiberberine | SCD |
| MOL002897 | epiberberine | AURKB |
| MOL002897 | epiberberine | XBP1 |
| MOL002897 | epiberberine | NTRK1 |
| MOL002897 | epiberberine | PIK3CD |
| MOL002897 | epiberberine | PIK3CB |
| MOL002897 | epiberberine | GRIA1 |
| MOL002897 | epiberberine | MAPKAPK2 |
| MOL002897 | epiberberine | JAK2 |
| MOL002897 | epiberberine | IMPDH2 |
| MOL002897 | epiberberine | CHEK2 |
| MOL002897 | epiberberine | TGM2 |
| MOL002897 | epiberberine | CD38 |
| MOL002897 | epiberberine | LCK |
| MOL002897 | epiberberine | PIM1 |
| MOL002897 | epiberberine | PIM2 |
| MOL002897 | epiberberine | MAPK10 |
| MOL002897 | epiberberine | MAP4K4 |
| MOL002897 | epiberberine | PARP1 |
| MOL002897 | epiberberine | F3 |
| MOL002897 | epiberberine | TRPM8 |
| MOL002897 | epiberberine | ABL1 |
| MOL002897 | epiberberine | SIRT3 |
| MOL002897 | epiberberine | SIRT2 |
| MOL002897 | epiberberine | SIRT1 |
| MOL002897 | epiberberine | NR3C2 |
| MOL002897 | epiberberine | DHFR |
| MOL002897 | epiberberine | FLT1 |
| MOL002897 | epiberberine | ALOX5AP |
| MOL002897 | epiberberine | ROCK1 |
| MOL002897 | epiberberine | BCAT2 |
| MOL002897 | epiberberine | PRKACA |
| MOL002897 | epiberberine | CYP11B2 |
| MOL002897 | epiberberine | ICAM1 |
| MOL002897 | epiberberine | SELE |
| MOL002897 | epiberberine | AGPAT2 |
| MOL002897 | epiberberine | DRD4 |
| MOL002897 | epiberberine | DRD3 |
| MOL002897 | epiberberine | KIT |
| MOL002897 | epiberberine | SRC |
| MOL002897 | epiberberine | MKNK1 |
| MOL002897 | epiberberine | FLT3 |
| MOL002897 | epiberberine | IKBKB |
| MOL002897 | epiberberine | ADORA2A |
| MOL002897 | epiberberine | ADORA3 |
| MOL002897 | epiberberine | PTPN1 |
| MOL002897 | epiberberine | ZAP70 |
| MOL002897 | epiberberine | SNCA |
| MOL002897 | epiberberine | JAK3 |
| MOL002897 | epiberberine | GSK3A |
| MOL002897 | epiberberine | IRAK4 |
| MOL002897 | epiberberine | MAPK1 |
| MOL002897 | epiberberine | SLC22A12 |
| MOL002897 | epiberberine | HTR3A |
| MOL002897 | epiberberine | AKR1B1 |
| MOL002897 | epiberberine | TYMS |
| MOL002897 | epiberberine | CHEK1 |
| MOL002897 | epiberberine | ERBB2 |
| MOL002897 | epiberberine | MAPK8 |
| MOL002897 | epiberberine | SCN9A |
| MOL002897 | epiberberine | NPY5R |
| MOL002897 | epiberberine | SLC5A1 |
| MOL002897 | epiberberine | PDE4B |
| MOL002897 | epiberberine | CCNE1 |
| MOL002897 | epiberberine | CYP11B1 |
| MOL002897 | epiberberine | EPHA2 |
| MOL002897 | epiberberine | FAAH |
| MOL002897 | epiberberine | MST1R |
| MOL002897 | epiberberine | DPP4 |
| MOL002897 | epiberberine | ROCK2 |
| MOL002897 | epiberberine | CDK8 |
| MOL002897 | epiberberine | CCNC |
| MOL002897 | epiberberine | MET |
| MOL002897 | epiberberine | STAT3 |
| MOL002897 | epiberberine | AXL |
| MOL002897 | epiberberine | MME |
| MOL002897 | epiberberine | RPS6KA3 |
| MOL002897 | epiberberine | EPHX2 |
| MOL002897 | epiberberine | QPCT |
| MOL002897 | epiberberine | MARS |
| MOL002897 | epiberberine | MMP9 |
| MOL002897 | epiberberine | NOS2 |
| MOL002897 | epiberberine | KCNH2 |
| MOL002897 | epiberberine | ESR1 |
| MOL002897 | epiberberine | AR |
| MOL002897 | epiberberine | PTGS2 |
| MOL002897 | epiberberine | RXRA |
| MOL002897 | epiberberine | PRSS1 |
| MOL002897 | epiberberine | NCOA2 |
| MOL002897 | epiberberine | PDE10A |
| MOL002897 | epiberberine | Bcl-2 |
| MOL002897 | epiberberine | Bax |
| MOL002897 | epiberberine | P53 |
| MOL002897 | epiberberine | CASP3 |
| MOL002268 | Rhein | FTO |
| MOL002268 | Rhein | CYP19A1 |
| MOL002268 | Rhein | ELANE |
| MOL002268 | Rhein | FNTA FNTB |
| MOL002268 | Rhein | CSNK2A1 |
| MOL002268 | Rhein | PTP4A3 |
| MOL002268 | Rhein | ESR2 |
| MOL002268 | Rhein | CASP3 |
| MOL002268 | Rhein | PIM1 |
| MOL002268 | Rhein | LDHA |
| MOL002268 | Rhein | LDHB |
| MOL002268 | Rhein | ERN1 |
| MOL002268 | Rhein | ESR1 |
| MOL002268 | Rhein | AMPD3 |
| MOL002268 | Rhein | BCL2 |
| MOL002268 | Rhein | ECE1 |
| MOL002268 | Rhein | CDC25B |
| MOL002268 | Rhein | MCL1 |
| MOL002268 | Rhein | LIMK1 |
| MOL002268 | Rhein | GRK6 |
| MOL002268 | Rhein | LCK |
| MOL002268 | Rhein | MME |
| MOL002268 | Rhein | CDK2 |
| MOL002268 | Rhein | HNF4A |
| MOL002268 | Rhein | F2 |
| MOL002268 | Rhein | IGFBP3 |
| MOL002268 | Rhein | EGLN1 |
| MOL002268 | Rhein | GPR35 |
| MOL002268 | Rhein | SLC13A5 |
| MOL002268 | Rhein | ACLY |
| MOL002268 | Rhein | CASP6 |
| MOL002268 | Rhein | CASP7 |
| MOL002268 | Rhein | CASP8 |
| MOL002268 | Rhein | CASP1 |
| MOL002268 | Rhein | CASP2 |
| MOL002268 | Rhein | OGA |
| MOL002268 | Rhein | MAPK8 |
| MOL002268 | Rhein | ADA |
| MOL002268 | Rhein | NOX4 |
| MOL002268 | Rhein | CAMKK2 |
| MOL002268 | Rhein | MMP16 |
| MOL002268 | Rhein | MMP13 |
| MOL002268 | Rhein | MMP9 |
| MOL002268 | Rhein | MMP1 |
| MOL002268 | Rhein | MMP2 |
| MOL002268 | Rhein | MMP14 |
| MOL002268 | Rhein | MMP8 |
| MOL002268 | Rhein | INSR |
| MOL002268 | Rhein | CDA |
| MOL002268 | Rhein | PTGS1 |
| MOL002268 | Rhein | PTGS2 |
| MOL002268 | Rhein | TNF |
| MOL002268 | Rhein | IL6 |
| MOL002268 | Rhein | HSP90AA1 |
| MOL002268 | Rhein | NCOA2 |
| MOL002268 | Rhein | AKR1B1 |
| MOL002268 | Rhein | JUN |
| MOL000472 | emodin | ESR1 |
| MOL000472 | emodin | TNF |
| MOL000472 | emodin | IL6 |
| MOL000472 | emodin | PIM1 |
| MOL000472 | emodin | ESR2 |
| MOL000472 | emodin | CSNK2A1 |
| MOL000472 | emodin | PTP4A3 |
| MOL000472 | emodin | ELANE |
| MOL000472 | emodin | FNTA |
| MOL000472 | emodin | MCL1 |
| MOL000472 | emodin | BCL2 |
| MOL000472 | emodin | FTO |
| MOL000472 | emodin | LIMK1 |
| MOL000472 | emodin | LCK |
| MOL000472 | emodin | CYP19A1 |
| MOL000472 | emodin | ABCB1 |
| MOL000472 | emodin | BCHE |
| MOL000472 | emodin | XDH |
| MOL000472 | emodin | ADORA3 |
| MOL000472 | emodin | CYP1B1 |
| MOL000472 | emodin | AURKB |
| MOL000472 | emodin | KDR |
| MOL000472 | emodin | PLK1 |
| MOL000472 | emodin | MET |
| MOL000472 | emodin | AXL |
| MOL000472 | emodin | EGFR |
| MOL000472 | emodin | FASN |
| MOL000472 | emodin | PARP1 |
| MOL000472 | emodin | TNKS2 |
| MOL000472 | emodin | TNKS |
| MOL000472 | emodin | CRHR1 |
| MOL000472 | emodin | DRD3 |
| MOL000472 | emodin | AKR1B1 |
| MOL000472 | emodin | CDK5R1 |
| MOL000472 | emodin | CCNB3 |
| MOL000472 | emodin | CDK6 |
| MOL000472 | emodin | ABCG2 |
| MOL000472 | emodin | CBR1 |
| MOL000472 | emodin | TBXAS1 |
| MOL000472 | emodin | MGAM |
| MOL000472 | emodin | HTR2C |
| MOL000472 | emodin | ESRRA |
| MOL000472 | emodin | ESRRB |
| MOL000472 | emodin | LDHA |
| MOL000472 | emodin | LDHB |
| MOL000472 | emodin | NOX4 |
| MOL000472 | emodin | FLT3 |
| MOL000472 | emodin | SYK |
| MOL000472 | emodin | GSK3B |
| MOL000472 | emodin | ABCC1 |
| MOL000472 | emodin | TTR |
| MOL000472 | emodin | CFTR |
| MOL000472 | emodin | AKR1B10 |
| MOL000472 | emodin | CXCR2 |
| MOL000472 | emodin | PTGS1 |
| MOL000472 | emodin | PTGS2 |
| MOL000472 | emodin | F7 |
| MOL000472 | emodin | HSP90AA1 |
| MOL000472 | emodin | PRKACA |
| MOL000472 | emodin | IGHG1 |
| MOL000472 | emodin | F10 |
| MOL000472 | emodin | TOP2A |
| MOL000472 | emodin | NCOA2 |
| MOL000472 | emodin | NCOA1 |
| MOL000472 | emodin | CAMKMT |
| MOL000472 | emodin | CDKN1A |
| MOL000472 | emodin | FLT1 |
| MOL000472 | emodin | MMP9 |
| MOL000472 | emodin | TNFAIP6 |
| MOL000472 | emodin | CASP3 |
| MOL000472 | emodin | TP53 |
| MOL000472 | emodin | PRKCE |
| MOL000472 | emodin | MMP1 |
| MOL000472 | emodin | PPARG |
| MOL000472 | emodin | MYC |
| MOL000472 | emodin | CYP1A1 |
| MOL000472 | emodin | IL1B |
| MOL000472 | emodin | PRKCD |
| MOL000472 | emodin | CSF2 |
| MOL000472 | emodin | ACTA2 |
| MOL000472 | emodin | MAOB |
| MOL000472 | emodin | BTK |
| MOL000472 | emodin | SLC2A4 |
| MOL000472 | emodin | FLT4 |
| MOL000472 | emodin | SLC2A1 |
| MOL001458 | coptisine | ACHE |
| MOL001458 | coptisine | SIGMAR1 |
| MOL001458 | coptisine | CHRM1 |
| MOL001458 | coptisine | HTR2B |
| MOL001458 | coptisine | BCHE |
| MOL001458 | coptisine | ADRA2C |
| MOL001458 | coptisine | ADRA2B |
| MOL001458 | coptisine | CYP2D6 |
| MOL001458 | coptisine | UBA2 |
| MOL001458 | coptisine | SAE1 |
| MOL001458 | coptisine | RAC1 |
| MOL001458 | coptisine | CDC42 |
| MOL001458 | coptisine | CHRM4 |
| MOL001458 | coptisine | TBXAS1 |
| MOL001458 | coptisine | XBP1 |
| MOL001458 | coptisine | IKBKB |
| MOL001458 | coptisine | PLK1 |
| MOL001458 | coptisine | PRF1 |
| MOL001458 | coptisine | ABL1 |
| MOL001458 | coptisine | CHEK2 |
| MOL001458 | coptisine | SCN9A |
| MOL001458 | coptisine | CDC7 |
| MOL001458 | coptisine | LIMK1 |
| MOL001458 | coptisine | SCD |
| MOL001458 | coptisine | DHFR |
| MOL001458 | coptisine | PIK3CD |
| MOL001458 | coptisine | PIK3CB |
| MOL001458 | coptisine | PIK3CG |
| MOL001458 | coptisine | CA2 |
| MOL001458 | coptisine | PARP1 |
| MOL001458 | coptisine | CSF1R |
| MOL001458 | coptisine | HPGD |
| MOL001458 | coptisine | RPS6KB1 |
| MOL001458 | coptisine | AURKA |
| MOL001458 | coptisine | TUBB1 |
| MOL001458 | coptisine | ADK |
| MOL001458 | coptisine | KIT |
| MOL001458 | coptisine | CYP19A1 |
| MOL001458 | coptisine | MAP3K8 |
| MOL001458 | coptisine | CDK8 |
| MOL001458 | coptisine | CCNC |
| MOL001458 | coptisine | PTGES |
| MOL001458 | coptisine | CDK8 |
| MOL001458 | coptisine | ADRA1D |
| MOL001458 | coptisine | FLT3 |
| MOL001458 | coptisine | MAOB |
| MOL001458 | coptisine | RPS27 |
| MOL001458 | coptisine | MAPK8 |
| MOL001458 | coptisine | SNCA |
| MOL001458 | coptisine | CYP11B1 |
| MOL001458 | coptisine | CYP11B2 |
| MOL001458 | coptisine | CHEK1 |
| MOL001458 | coptisine | BRAF |
| MOL001458 | coptisine | MME |
| MOL001458 | coptisine | LRRK2 |
| MOL001458 | coptisine | IMPDH2 |
| MOL001458 | coptisine | CYP17A1 |
| MOL001458 | coptisine | MCL1 |
| MOL001458 | coptisine | ALOX5AP |
| MOL001458 | coptisine | CDK2 |
| MOL001458 | coptisine | CCNA2 |
| MOL001458 | coptisine | CCNA1 |
| MOL001458 | coptisine | AURKB |
| MOL001458 | coptisine | PTGS1 |
| MOL001458 | coptisine | PTGS2 |
| MOL001458 | coptisine | PDE4B |
| MOL001458 | coptisine | ERBB2 |
| MOL001458 | coptisine | ATP4A |
| MOL001458 | coptisine | ATP4B |
| MOL001458 | coptisine | PDE4A |
| MOL001458 | coptisine | ADORA2B |
| MOL001458 | coptisine | MAPKAPK2 |
| MOL001458 | coptisine | PYGL |
| MOL001458 | coptisine | HSD11B1 |
| MOL001458 | coptisine | CHRM5 |
| MOL001458 | coptisine | ENPP1 |
| MOL001458 | coptisine | PDGFRB |
| MOL001458 | coptisine | FLT4 |
| MOL001458 | coptisine | SRC |
| MOL001458 | coptisine | TNNI3K |
| MOL001458 | coptisine | RPS6KA3 |
| MOL001458 | coptisine | MYLK |
| MOL001458 | coptisine | DCK |
| MOL001458 | coptisine | TUBB3 |
| MOL001458 | coptisine | PLK4 |
| MOL001458 | coptisine | ABCB1 |
| MOL001458 | coptisine | AKR1B1 |
| MOL001458 | coptisine | PIK3R1 |
| MOL001458 | coptisine | PIK3CA |
| MOL001458 | coptisine | ADORA3 |
| MOL001458 | coptisine | AKT2 |
| MOL001458 | coptisine | PDPK1 |
| MOL001458 | coptisine | PRKCA |
| MOL001458 | coptisine | MAP2K1 |
| MOL001458 | coptisine | JAK3 |
| MOL001458 | coptisine | LCK |
| MOL001458 | coptisine | TGM2 |
| MOL001458 | coptisine | NTRK1 |
| MOL001458 | coptisine | GSK3A |
| MOL001458 | coptisine | ITK |
| MOL001458 | coptisine | STS |
| MOL001458 | coptisine | MET |
| MOL001458 | coptisine | IRAK4 |
| MOL001458 | coptisine | MAPK1 |
| MOL001458 | coptisine | HSD17B3 |
| MOL001458 | coptisine | PHLPP2 |
| MOL001458 | coptisine | NOS2 |
| MOL001458 | coptisine | KCNH2 |
| MOL001458 | coptisine | ESR1 |
| MOL001458 | coptisine | AR |
| MOL001458 | coptisine | SCN5A |
| MOL001458 | coptisine | PRSS1 |
| MOL001458 | coptisine | IL6 |
| MOL001458 | coptisine | TNF |
| MOL001458 | coptisine | CASP3 |
| MOL001458 | coptisine | KCNH2 |
| MOL001458 | coptisine | GPER1 |
| MOL001458 | coptisine | NCOA4 |
| MOL001458 | coptisine | eNOS |
| MOL001458 | coptisine | TRY1 |
| MOL000476 | Physcion | ELANE |
| MOL000476 | Physcion | LIMK1 |
| MOL000476 | Physcion | PTP4A3 |
| MOL000476 | Physcion | CSNK2A1 |
| MOL000476 | Physcion | LCK |
| MOL000476 | Physcion | ESR1 |
| MOL000476 | Physcion | ESR2 |
| MOL000476 | Physcion | PIM1 |
| MOL000476 | Physcion | MCL1 |
| MOL000476 | Physcion | DUSP3 |
| MOL000476 | Physcion | EGFR |
| MOL000476 | Physcion | BCL2 |
| MOL000476 | Physcion | FTO |
| MOL000476 | Physcion | EIF2AK2 |
| MOL000476 | Physcion | MME |
| MOL000476 | Physcion | MMP3 |
| MOL000476 | Physcion | MMP1 |
| MOL000476 | Physcion | MMP9 |
| MOL000476 | Physcion | MMP2 |
| MOL000476 | Physcion | MMP8 |
| MOL000476 | Physcion | PDE5A |
| MOL000476 | Physcion | NQO1 |
| MOL000476 | Physcion | MMP13 |
| MOL000476 | Physcion | CHRNA7 |
| MOL000476 | Physcion | HDAC6 |
| MOL000476 | Physcion | HDAC8 |
| MOL000476 | Physcion | HDAC1 |
| MOL000476 | Physcion | MMP7 |
| MOL000476 | Physcion | MMP10 |
| MOL000476 | Physcion | KDM1A |
| MOL000476 | Physcion | ADAM17 |
| MOL000476 | Physcion | MAPK8 |
| MOL000476 | Physcion | MMP16 |
| MOL000476 | Physcion | MMP14 |
| MOL000476 | Physcion | KCNMA1 |
| MOL000476 | Physcion | MMP12 |
| MOL000476 | Physcion | CTSV |
| MOL000476 | Physcion | BAD |
| MOL000476 | Physcion | TERT |
| MOL000476 | Physcion | MAP2K1 |
| MOL000476 | Physcion | CYP19A1 |
| MOL000476 | Physcion | CYP1B1 |
| MOL000476 | Physcion | NOX4 |
| MOL000476 | Physcion | BCHE |
| MOL000476 | Physcion | PLA2G7 |
| MOL000476 | Physcion | GUSB |
| MOL000476 | Physcion | FLT3 |
| MOL000476 | Physcion | NOTUM |
| MOL000476 | Physcion | LRRK2 |
| MOL000476 | Physcion | HDAC5 |
| MOL000476 | Physcion | HDAC7 |
| MOL000476 | Physcion | HDAC4 |
| MOL000476 | Physcion | HDAC9 |
| MOL000476 | Physcion | ADAMTS5 |
| MOL000476 | Physcion | ADAMTS4 |
| MOL000476 | Physcion | PDE4B |
| MOL000476 | Physcion | PDE4C |
| MOL000476 | Physcion | MET |
| MOL000476 | Physcion | ANPEP |
| MOL000476 | Physcion | PIK3CD |
| MOL000476 | Physcion | CCNE1 |
| MOL000476 | Physcion | ERBB2 |
| MOL000476 | Physcion | FLT1 |
| MOL000476 | Physcion | PDGFRB |
| MOL000476 | Physcion | FLT4 |
| MOL000476 | Physcion | PDGFRA |
| MOL000476 | Physcion | KDR |
| MOL000476 | Physcion | GRK6 |
| MOL000476 | Physcion | PTGS1 |
| MOL000476 | Physcion | SCN5A |
| MOL000476 | Physcion | PTGS2 |
| MOL000476 | Physcion | F7 |
| MOL000476 | Physcion | TOP2A |
| MOL000476 | Physcion | HSP90AA1 |
| MOL000476 | Physcion | DPEP1 |
| MOL000476 | Physcion | IGHG1 |
| MOL000476 | Physcion | NCOA2 |
| MOL000476 | Physcion | NCOA1 |
| MOL000476 | Physcion | PKIA |
| MOL000476 | Physcion | CAMKMT |
| MOL000476 | Physcion | PRKACA |
| MOL000476 | Physcion | F10 |
| MOL000476 | Physcion | RXRA |
| MOL000476 | Physcion | CASP3 |
| MOL000476 | Physcion | TNF |
| MOL000476 | Physcion | IL6 |
| MOL003305 | PHILLYRIN | ADORA1 |
| MOL003305 | PHILLYRIN | MCL1 |
| MOL003305 | PHILLYRIN | TDP1 |
| MOL003305 | PHILLYRIN | SOAT1 |
| MOL003305 | PHILLYRIN | HIF1A |
| MOL003305 | PHILLYRIN | SOAT2 |
| MOL003305 | PHILLYRIN | SRD5A1 |
| MOL003305 | PHILLYRIN | SLC5A2 |
| MOL003305 | PHILLYRIN | IL6 |
| MOL003305 | PHILLYRIN | TNF |
| MOL003305 | PHILLYRIN | CASP3 |
| MOL003137 | Leucanthoside | TOP2A |
| MOL003137 | Leucanthoside | PTPN1 |
| MOL006554 | Taraxerol | AKR1C1 |
| MOL006554 | Taraxerol | AKR1C2 |
| MOL006554 | Taraxerol | AR |
| MOL006554 | Taraxerol | CLEC4E |
| MOL006554 | Taraxerol | ESR1 |
| MOL006554 | Taraxerol | ESR2 |
| MOL006554 | Taraxerol | GABRA1 |
| MOL006554 | Taraxerol | GABRA2 |
| MOL006554 | Taraxerol | GABRA3 |
| MOL006554 | Taraxerol | GABRA4 |
| MOL006554 | Taraxerol | GABRA5 |
| MOL006554 | Taraxerol | GABRA6 |
| MOL006554 | Taraxerol | GABRB1 |
| MOL006554 | Taraxerol | GABRB2 |
| MOL006554 | Taraxerol | GABRB3 |
| MOL006554 | Taraxerol | GABRD |
| MOL006554 | Taraxerol | GABRE |
| MOL006554 | Taraxerol | GABRG1 |
| MOL006554 | Taraxerol | GABRG2 |
| MOL006554 | Taraxerol | GABRG3 |
| MOL006554 | Taraxerol | GABRP |
| MOL006554 | Taraxerol | GABRQ |
| MOL006554 | Taraxerol | GRIN1 |
| MOL006554 | Taraxerol | GRIN2A |
| MOL006554 | Taraxerol | GRIN2B |
| MOL006554 | Taraxerol | GRIN2C |
| MOL006554 | Taraxerol | GRIN2D |
| MOL006554 | Taraxerol | GRIN3A |
| MOL006554 | Taraxerol | GRIN3B |
| MOL006554 | Taraxerol | HSD17B1 |
| MOL006554 | Taraxerol | LSS |
| MOL006554 | Taraxerol | NCOA2 |
| MOL006554 | Taraxerol | NFKB1 |
| MOL006554 | Taraxerol | NFKB2 |
| MOL006554 | Taraxerol | NR1I2 |
| MOL006554 | Taraxerol | NR1I3 |
| MOL006554 | Taraxerol | NR3C1 |
| MOL006554 | Taraxerol | NR3C2 |
| MOL006554 | Taraxerol | PGR |
| MOL006554 | Taraxerol | PPARA |
| MOL006554 | Taraxerol | RORA |
| MOL006554 | Taraxerol | SHBG |
| MOL006554 | Taraxerol | SIGMAR1 |
| MOL006554 | Taraxerol | SULT2A1 |
| MOL006554 | Taraxerol | SULT2B1 |
| MOL006554 | Taraxerol | VDR |
