# Supplementary material for: Tanhuo Formula Inhibits Astrocyte Activation and Apoptosis in Acute Ischemic Stroke
Source: Front Pharmacol. 2022 Apr 26;13:859244. doi: 10.3389/fphar.2022.859244 (PMC9087855; doi:10.3389/fphar.2022.859244)
Supplement: Supplementary file 3 [file Table3.DOCX]

**Supplementary Table S3：Overlapped genes**

| No. | Symbol name | BetweennessCentrality | ClosenessCentrality | Degree |
| --- | --- | --- | --- | --- |
| 1 | AKT1 | 115 | 0.08233976 | 0.78217822 |
| 2 | TNF | 112 | 0.05964128 | 0.76699029 |
| 3 | IL6 | 110 | 0.05653145 | 0.76328502 |
| 4 | IL1B | 101 | 0.03363536 | 0.73148148 |
| 5 | CASP3 | 94 | 0.03566401 | 0.70535714 |
| 6 | TP53 | 93 | 0.02258106 | 0.70222222 |
| 7 | FN1 | 88 | 0.02324471 | 0.68695652 |
| 8 | EGFR | 86 | 0.02209445 | 0.68103448 |
| 9 | SRC | 84 | 0.02334333 | 0.67234043 |
| 10 | MMP9 | 84 | 0.01850454 | 0.66666667 |
| 11 | JUN | 83 | 0.01728606 | 0.66949153 |
| 12 | STAT3 | 81 | 0.01342416 | 0.66108787 |
| 13 | HIF1A | 81 | 0.01146068 | 0.66666667 |
| 14 | PTGS2 | 77 | 0.01059601 | 0.65020576 |
| 15 | CCL2 | 77 | 0.00926775 | 0.64754098 |
| 16 | CXCL8 | 77 | 0.01175682 | 0.64754098 |
| 17 | MYC | 75 | 0.01422536 | 0.64489796 |
| 18 | NOS3 | 74 | 0.03387654 | 0.65020576 |
| 19 | ESR1 | 71 | 0.01457001 | 0.63967611 |
| 20 | FOS | 71 | 0.02090285 | 0.63967611 |
| 21 | PPARG | 70 | 0.01043437 | 0.63453815 |
| 22 | MTOR | 66 | 0.00927812 | 0.62698413 |
| 23 | MMP2 | 66 | 0.00530661 | 0.61960784 |
| 24 | ICAM1 | 65 | 0.00483785 | 0.61478599 |
| 25 | ERBB2 | 63 | 0.00717291 | 0.6124031 |
| 26 | ACE | 61 | 0.01875705 | 0.61003861 |
| 27 | SERPINE1 | 61 | 0.0087514 | 0.60305344 |
| 28 | VCAM1 | 61 | 0.00491048 | 0.60305344 |
| 29 | IL4 | 61 | 0.00409295 | 0.60536398 |
| 30 | APP | 60 | 0.0198972 | 0.61003861 |
| 31 | MAPK8 | 59 | 0.00613885 | 0.60536398 |
| 32 | IFNG | 59 | 0.00348912 | 0.60076046 |
| 33 | BCL2L1 | 57 | 0.00289511 | 0.59622642 |
| 34 | MAPK1 | 56 | 0.00780738 | 0.59848485 |
| 35 | RELA | 55 | 0.00198034 | 0.58955224 |
| 36 | HMOX1 | 55 | 0.00448394 | 0.59848485 |
| 37 | MAPK14 | 55 | 0.0032496 | 0.59398496 |
| 38 | SIRT1 | 54 | 0.00822214 | 0.59398496 |
| 39 | STAT1 | 53 | 0.00315972 | 0.58518519 |
| 40 | KDR | 52 | 0.00344233 | 0.59398496 |
| 41 | SPP1 | 49 | 0.00513372 | 0.58088235 |
| 42 | HSPA5 | 48 | 0.00597518 | 0.58088235 |
| 43 | MPO | 47 | 0.00457331 | 0.57039711 |
| 44 | MAP2K1 | 46 | 0.00303078 | 0.57039711 |
| 45 | CASP9 | 44 | 0.00119899 | 0.56834532 |
| 46 | MMP1 | 44 | 9.11E-04 | 0.56630824 |
| 47 | SELE | 43 | 8.70E-04 | 0.56028369 |
| 48 | CASP1 | 43 | 0.00114807 | 0.56227758 |
| 49 | JAK2 | 43 | 9.86E-04 | 0.56227758 |
| 50 | PPARA | 42 | 0.00157966 | 0.56630824 |
| 51 | MMP3 | 41 | 8.45E-04 | 0.56028369 |
| 52 | PIK3CA | 41 | 0.00246217 | 0.54861111 |
| 53 | IL1A | 41 | 6.27E-04 | 0.55830389 |
| 54 | NOS2 | 41 | 0.00283532 | 0.56630824 |
| 55 | F2 | 40 | 0.00726486 | 0.56028369 |
| 56 | CXCL10 | 39 | 0.00106744 | 0.55052265 |
| 57 | PLAU | 39 | 8.37E-04 | 0.55244755 |
| 58 | NFE2L2 | 37 | 8.55E-04 | 0.55438596 |
| 59 | NR3C1 | 36 | 0.00274993 | 0.55438596 |
| 60 | PDGFRB | 36 | 0.00148423 | 0.5467128 |
| 61 | XIAP | 35 | 6.08E-04 | 0.54482759 |
| 62 | HSPB1 | 35 | 0.00151482 | 0.55244755 |
| 63 | SOD1 | 35 | 0.01104978 | 0.54861111 |
| 64 | CD40LG | 35 | 0.0016369 | 0.54482759 |
| 65 | SNCA | 34 | 0.00668901 | 0.55052265 |
| 66 | RAF1 | 34 | 0.00160772 | 0.53924915 |
| 67 | MMP7 | 34 | 4.26E-04 | 0.5467128 |
| 68 | FLT1 | 34 | 7.35E-04 | 0.5467128 |
| 69 | F3 | 33 | 0.00144898 | 0.53741497 |
| 70 | NTRK1 | 33 | 0.01206447 | 0.54861111 |
| 71 | MUC1 | 32 | 0.00102449 | 0.53924915 |
| 72 | GRIN2B | 32 | 0.01054663 | 0.54482759 |
| 73 | GJA1 | 31 | 0.01355108 | 0.54861111 |
| 74 | TEK | 30 | 8.03E-04 | 0.53924915 |
| 75 | IGFBP3 | 30 | 9.67E-04 | 0.53559322 |
| 76 | ABCB1 | 29 | 0.00190402 | 0.54295533 |
| 77 | NCF1 | 29 | 5.44E-04 | 0.53378378 |
| 78 | SLC2A1 | 28 | 0.00451355 | 0.54109589 |
| 79 | PLAT | 28 | 0.00211751 | 0.52666667 |
| 80 | CSF1R | 28 | 6.86E-04 | 0.53198653 |
| 81 | PARP1 | 28 | 2.44E-04 | 0.53198653 |
| 82 | CTSD | 27 | 5.45E-04 | 0.53020134 |
| 83 | ACTA2 | 27 | 0.00101545 | 0.52842809 |
| 84 | GRM5 | 27 | 0.00743424 | 0.53020134 |
| 85 | MMP13 | 27 | 2.81E-04 | 0.53198653 |
| 86 | BAX | 26 | 3.91E-04 | 0.52491694 |
| 87 | ELANE | 26 | 1.98E-04 | 0.51973684 |
| 88 | THBD | 25 | 6.90E-04 | 0.50479233 |
| 89 | TERT | 25 | 2.20E-04 | 0.52666667 |
| 90 | LRRK2 | 24 | 0.00116415 | 0.51973684 |
| 91 | DPP4 | 24 | 0.00102931 | 0.52842809 |
| 92 | GRIN1 | 24 | 0.00667319 | 0.52666667 |
| 93 | MMP8 | 24 | 1.11E-04 | 0.52317881 |
| 94 | GRIA1 | 23 | 0.00573892 | 0.51132686 |
| 95 | PIK3CG | 23 | 0.00179619 | 0.51633987 |
| 96 | MAPK10 | 23 | 0.00160287 | 0.52145215 |
| 97 | IL6R | 22 | 1.07E-04 | 0.51633987 |
| 98 | SLC6A4 | 20 | 0.00699086 | 0.51132686 |
| 99 | ADRB2 | 20 | 0.00127135 | 0.52491694 |
| 100 | BCL2 | 20 | 4.54E-04 | 0.49375 |
| 101 | ADORA1 | 19 | 0.00609628 | 0.52145215 |
| 102 | PRKCE | 19 | 0.01374698 | 0.49529781 |
| 103 | BRAF | 19 | 4.50E-04 | 0.5 |
| 104 | ALOX5 | 19 | 5.17E-04 | 0.50967742 |
| 105 | GRIN2A | 19 | 0.00255037 | 0.51132686 |
| 106 | MME | 18 | 7.53E-04 | 0.50318471 |
| 107 | SLC6A3 | 18 | 0.00174185 | 0.50318471 |
| 108 | COL3A1 | 18 | 4.11E-04 | 0.48318043 |
| 109 | ACHE | 17 | 8.74E-04 | 0.51803279 |
| 110 | GRIA2 | 17 | 0.00250818 | 0.47590361 |
| 111 | ADORA2A | 17 | 0.00468482 | 0.51633987 |
| 112 | GRM1 | 17 | 0.00251455 | 0.48916409 |
| 113 | RASA1 | 17 | 5.83E-04 | 0.48765432 |
| 114 | PTGS1 | 16 | 4.94E-04 | 0.5015873 |
| 115 | MAP2 | 16 | 0.00107893 | 0.49842271 |
| 116 | BAD | 16 | 2.50E-04 | 0.48170732 |
| 117 | PON1 | 15 | 5.07E-04 | 0.48916409 |
| 118 | CRH | 15 | 0.00129385 | 0.5015873 |
| 119 | DRD1 | 15 | 0.00111047 | 0.44632768 |
| 120 | TUBB3 | 14 | 6.43E-04 | 0.49375 |
| 121 | ADORA3 | 14 | 0.00346748 | 0.50967742 |
| 122 | ADRBK1 | 14 | 0.00156262 | 0.49221184 |
| 123 | PPP3CA | 14 | 6.85E-04 | 0.49221184 |
| 124 | SLC1A3 | 14 | 0.00113616 | 0.48466258 |
| 125 | HMGCR | 13 | 0.00117248 | 0.5015873 |
| 126 | TTR | 13 | 7.84E-04 | 0.49375 |
| 127 | NPPB | 13 | 5.79E-04 | 0.49375 |
| 128 | ADA | 13 | 0.00307173 | 0.49068323 |
| 129 | F7 | 12 | 2.55E-04 | 0.47305389 |
| 130 | BCHE | 11 | 4.82E-04 | 0.48765432 |
| 131 | VCP | 11 | 1.19E-04 | 0.48170732 |
| 132 | GABRG2 | 11 | 0.00187239 | 0.40306122 |
| 133 | NR3C2 | 10 | 7.21E-04 | 0.47164179 |
| 134 | MYLK | 10 | 4.53E-04 | 0.49685535 |
| 135 | XDH | 10 | 2.26E-04 | 0.48318043 |
| 136 | GABRA1 | 10 | 1.39E-04 | 0.3872549 |
| 137 | EPHB4 | 10 | 2.18E-04 | 0.47305389 |
| 138 | ENPP1 | 9 | 0.00111326 | 0.42818428 |
| 139 | AOC3 | 9 | 0.00183479 | 0.45797101 |
| 140 | EIF2AK2 | 9 | 1.56E-05 | 0.47878788 |
| 141 | PDE5A | 8 | 9.17E-04 | 0.48170732 |
| 142 | ADRB1 | 8 | 5.21E-04 | 0.48466258 |
| 143 | ODC1 | 8 | 5.22E-06 | 0.46745562 |
| 144 | F10 | 8 | 0 | 0.4566474 |
| 145 | DHFR | 7 | 0.00139977 | 0.46884273 |
| 146 | ADK | 7 | 5.14E-04 | 0.36658933 |
| 147 | DYRK1A | 7 | 4.94E-05 | 0.47164179 |
| 148 | SHBG | 7 | 6.91E-05 | 0.4566474 |
| 149 | PLA2G7 | 6 | 1.44E-05 | 0.4619883 |
| 150 | PDE3A | 5 | 7.17E-04 | 0.45402299 |
| 151 | KCNMA1 | 5 | 0.00256818 | 0.46334311 |
| 152 | ALOX5AP | 5 | 1.38E-05 | 0.44759207 |
| 153 | CYP11B2 | 4 | 2.69E-05 | 0.41361257 |
| 154 | SCN9A | 4 | 6.88E-04 | 0.37089202 |
| 155 | ALDH2 | 3 | 1.02E-04 | 0.37889688 |
| 156 | SCN5A | 3 | 1.94E-04 | 0.36238532 |
| 157 | CA2 | 2 | 0 | 0.41798942 |
| 158 | LIMK1 | 2 | 6.72E-06 | 0.42934783 |
| 159 | ADRA2B | 1 | 0 | 0.33193277 |
