# Supplementary material for: Tanhuo Formula Inhibits Astrocyte Activation and Apoptosis in Acute Ischemic Stroke
Source: Front Pharmacol. 2022 Apr 26;13:859244. doi: 10.3389/fphar.2022.859244 (PMC9087855; doi:10.3389/fphar.2022.859244)
Supplement: Supplementary file 4 [file Table4.DOCX]

**Supplementary Table S4：Top 20 of KEGG pathway analysis based on 159 overlapping genes**

| No. | Pathway ID | Pathway Name | P value | Count | Gene Name |
| --- | --- | --- | --- | --- | --- |
| 1 | hsa04933 | AGE-RAGE signaling pathway in diabetic complications | 6.98E-28 | 31 | BCL2/BAX/JUN/CASP3/PRKCE/IL1B/IL6/TNF/RELA/AKT1/MMP2/MAPK1/STAT1/F3/ICAM1/CCL2/SELE/VCAM1/CXCL8/NOS3/THBD/SERPINE1/IL1A/COL3A1/MAPK14/FN1/MAPK8/PIK3CA/MAPK10/JAK2/STAT3 |
| 2 | hsa05417 | Lipid and atherosclerosis | 4.37E-25 | 38 | BCL2/BAX/CASP9/JUN/CASP3/TP53/IL1B/IL6/TNF/PPARG/MMP3/RELA/AKT1/BCL2L1/FOS/MMP9/MAPK1/MMP1/HSPA5/ICAM1/CCL2/SELE/VCAM1/CXCL8/NOS3/NCF1/NFE2L2/CD40LG/MAPK14/MAPK8/PPP3CA/SRC/PIK3CA/MAPK10/JAK2/STAT3/CASP1/BAD |
| 3 | hsa05418 | Fluid shear stress and atherosclerosis | 9.26E-20 | 28 | KDR/BCL2/JUN/TP53/IL1B/TNF/RELA/AKT1/FOS/MMP2/MMP9/HMOX1/ICAM1/CCL2/SELE/VCAM1/NOS3/PLAT/THBD/IFNG/IL1A/NCF1/NFE2L2/MAPK14/MAPK8/SRC/PIK3CA/MAPK10 |
| 4 | hsa05161 | Hepatitis B | 5.93E-17 | 27 | BCL2/BAX/CASP9/JUN/CASP3/TP53/MYC/IL6/TNF/RELA/AKT1/FOS/MMP9/MAPK1/RAF1/STAT1/CXCL8/MAPK14/MAPK8/MAP2K1/SRC/PIK3CA/MAPK10/JAK2/STAT3/BRAF/BAD |
| 5 | hsa05167 | Kaposi sarcoma-associated herpesvirus infection | 5.93E-17 | 29 | PTGS2/BAX/CASP9/JUN/CASP3/TP53/MYC/IL6/RELA/AKT1/FOS/MAPK1/RAF1/HIF1A/STAT1/ICAM1/CXCL8/MAPK14/MAPK8/PPP3CA/PIK3CG/MAP2K1/SRC/MTOR/PIK3CA/EIF2AK2/MAPK10/JAK2/STAT3 |
| 6 | hsa01522 | Endocrine resistance | 1.22E-16 | 22 | ESR1/BCL2/BAX/JUN/TP53/EGFR/AKT1/FOS/MMP2/MMP9/MAPK1/RAF1/ERBB2/MAPK14/MAPK8/MAP2K1/SRC/MTOR/PIK3CA/MAPK10/BRAF/BAD |
| 7 | hsa01521 | EGFR tyrosine kinase inhibitor resistance | 3.04E-16 | 20 | KDR/BCL2/BAX/IL6/EGFR/AKT1/BCL2L1/MAPK1/IL6R/RAF1/ERBB2/MAP2K1/SRC/MTOR/PIK3CA/JAK2/STAT3/PDGFRB/BRAF/BAD |
| 8 | hsa04657 | IL-17 signaling pathway | 6.29E-16 | 21 | PTGS2/JUN/CASP3/IL1B/IL6/TNF/MMP3/RELA/FOS/MMP9/MAPK1/MMP1/CCL2/CXCL8/IFNG/CXCL10/MAPK14/MAPK8/IL4/MMP13/MAPK10 |
| 9 | hsa04668 | TNF signaling pathway | 1.73E-15 | 22 | PTGS2/JUN/CASP3/IL1B/IL6/TNF/MMP3/RELA/AKT1/FOS/MMP9/MAPK1/ICAM1/CCL2/SELE/VCAM1/CXCL10/MAPK14/MAPK8/MAP2K1/PIK3CA/MAPK10 |
| 10 | hsa05212 | Pancreatic cancer | 1.92E-15 | 19 | BAX/CASP9/TP53/RELA/EGFR/AKT1/BCL2L1/MAPK1/RAF1/STAT1/ERBB2/MAPK8/MAP2K1/MTOR/PIK3CA/MAPK10/STAT3/BRAF/BAD |
| 11 | hsa04066 | HIF-1 signaling pathway | 1.16E-14 | 21 | NOS2/BCL2/IL6/RELA/EGFR/AKT1/MAPK1/IL6R/HIF1A/ERBB2/HMOX1/NOS3/SERPINE1/IFNG/MAP2K1/MTOR/PIK3CA/TEK/STAT3/FLT1/SLC2A1 |
| 12 | hsa05145 | Toxoplasmosis | 1.80E-14 | 21 | NOS2/BCL2/CASP9/CASP3/TNF/RELA/AKT1/BCL2L1/MAPK1/STAT1/IFNG/ALOX5/CD40LG/MAPK14/MAPK8/XIAP/PIK3CG/MAPK10/JAK2/STAT3/BAD |
| 13 | hsa05210 | Colorectal cancer | 1.80E-14 | 19 | BCL2/BAX/CASP9/JUN/CASP3/TP53/MYC/EGFR/AKT1/FOS/MAPK1/RAF1/MAPK8/MAP2K1/MTOR/PIK3CA/MAPK10/BRAF/BAD |
| 14 | hsa04210 | Apoptosis | 8.30E-14 | 22 | BCL2/BAX/CASP9/JUN/CASP3/TP53/TNF/RELA/AKT1/BCL2L1/FOS/MAPK1/RAF1/PARP1/CTSD/MAPK8/XIAP/MAP2K1/PIK3CA/MAPK10/NTRK1/BAD |
| 15 | hsa05215 | Prostate cancer | 1.68E-13 | 19 | BCL2/CASP9/TP53/PLAU/MMP3/RELA/EGFR/AKT1/MMP9/MAPK1/RAF1/ERBB2/PLAT/MAP2K1/MTOR/PIK3CA/PDGFRB/BRAF/BAD |
| 16 | hsa04926 | Relaxin signaling pathway | 2.80E-13 | 21 | NOS2/JUN/RELA/EGFR/AKT1/FOS/MMP2/MMP9/MAPK1/RAF1/MMP1/NOS3/COL3A1/MAPK14/MAPK8/MMP13/MAP2K1/SRC/PIK3CA/MAPK10/ACTA2 |
| 17 | hsa05022 | Pathways of neurodegeneration - multiple diseases | 6.45E-13 | 37 | NOS2/JUN/RELA/EGFR/AKT1/FOS/MMP2/MMP9/MAPK1/RAF1/MMP1/NOS3/COL3A1/MAPK14/MAPK8/MMP13/MAP2K1/SRC/PIK3CA/MAPK10/ACTA2 |
| 18 | hsa05152 | Tuberculosis | 2.77E-12 | 23 | NOS2/BCL2/BAX/CASP9/CASP3/IL1B/IL6/TNF/RELA/AKT1/MAPK1/RAF1/STAT1/IFNG/IL1A/CTSD/MAPK14/MAPK8/PPP3CA/SRC/MAPK10/JAK2/BAD |
| 19 | hsa05219 | Bladder cancer | 3.18E-12 | 13 | TP53/MYC/EGFR/MMP2/MMP9/MAPK1/RAF1/MMP1/ERBB2/CXCL8/MAP2K1/SRC/BRAF |
| 20 | hsa04024 | cAMP signaling pathway | 3.43E-12 | 25 | DRD1/PDE3A/ADRB2/JUN/ADRB1/GRIA2/RELA/AKT1/FOS/MAPK1/RAF1/PPARA/MAPK8/CRH/MAP2K1/PIK3CA/GRIA1/MAPK10/ADORA2A/BRAF/BAD/ADORA1/GRIN1/GRIN2A/GRIN2B |
